# Hormonal contraceptive use and *Staphylococcus aureus* nasal and throat carriage in a Norwegian youth population

**DOI:** 10.1371/journal.pone.0218511

**Published:** 2019-07-05

**Authors:** Dina B. Stensen, Lars Småbrekke, Karina Olsen, Guri Grimnes, Christopher Sivert Nielsen, Gunnar Skov Simonsen, Johanna U. E. Sollid, Anne-Sofie Furberg

**Affiliations:** 1 Department of Community Medicine, Faculty of Health Sciences, UiT The Arctic University of Norway, Tromsø, Norway; 2 Division of Internal Medicine, University Hospital of North Norway, Tromsø, Norway; 3 Department of Pharmacy, Faculty of Health Sciences, UiT The Arctic University of Norway, Tromsø, Norway; 4 Department of Microbiology and Infection Control, University Hospital of North Norway, Tromsø, Norway; 5 Endocrinology Research Group, Department of Clinical Medicine, Faculty of Health Sciences, UiT The Arctic University of Norway, Tromsø, Norway; 6 Division of Ageing and Health, Norwegian Institute of Public Health, Oslo, Norway; 7 Department of Pain Management and Research, Division of Emergencies and Intensive Care, Oslo University Hospital, Oslo, Norway; 8 Research Group for Host-Microbe Interaction, Department of Medical Biology, Faculty of Health Sciences, UiT The Arctic University of Norway, Tromsø, Norway; Laurentian, CANADA

## Abstract

**Background:**

Use of hormonal contraceptives has been associated with *Staphylococcus aureus* nasal carriage in adult women. However, the role of hormonal contraceptives in *S*. *aureus* colonization among adolescents and associations with progestin only contraceptives are unknown.

**Methods:**

We obtained nasal and throat swab samples from 439 girls aged 17–21 years in the population-based Tromsø study Fit Futures, 2012–2013, Norway, with information on lifestyle, health and biomarkers. We used multivariable logistic regression to study the association between use of hormonal contraceptives and *Staphylococcus aureus* carriage while adjusting for potential confounding factors.

**Results:**

*Staphylococcus aureus* nasal carriage prevalence were 34%, 42%, and 61% among progestin-only users, non-users, and progestin-estrogen combination contraceptive users, respectively (P<0.001). Use of combination contraceptives doubled the odds of nasal carriage (non-users reference; OR = 2.31, 95%CI = 1.43–3.74). The OR of nasal carriage was 0.29 among progestin-only users compared to combination contraceptives users (95% CI = 0.12–0.67).

**Discussion:**

In this study, use of combination hormonal contraceptives was associated with higher risk of *Staphylococcus aureus* nasal carriage in adolescent girls. Experimental design studies are needed to establish the role of exogenous sex steroids in *Staphylococcus aureus* colonization in women.

## Introduction

*Staphylococcus aureus* colonizes the skin and mucosal surfaces including nose and throat, and may cause a wide range of clinically important infections [1–3]. The nasal mucosa is the major *S*. *aureus* reservoir associated with transmission to other body sites and auto-infections, as well as transmission to others [1, 3–4]. The prevalence of nasal carriage increases from 20–30% in young children to 40–50% in older children and adolescents, after which the prevalence drops to 20–30% in the adult population [1, 3, 5–6]. Men have higher *S*. *aureus* nasal carrier rates than women [7]. Exclusive throat carriage is increasingly identified as an additional *S*. *aureus* reservoir particularly in young populations, but is considered less important in transmission and infection [8–11].

Prevention and eradication of *S*. *aureus* carriage may reduce the *S*. *aureus* disease burden [1, 3, 12]. In the carrier state, *S*. *aureus* is not successfully cleared by the host innate immune system, which function is determined by genes and environment. No significant heritability of *S*. *aureus* nasal carriage was found in a twin study [13], and evidence for host genetic determinants from observational studies is scarce [14–18]. This motivates the search for modifiable host lifestyle and environmental determinants as potential targets for prevention and infection control [4]. The strong associations of *S*. *aureus* carriage with age and sex, suggest that reproductive hormones may be key factors in regulating the immune response. Both endogenous and exogenous sex steroid exposure have been associated with *S*. *aureus* nasal carriage in women [19–21]. The initial hypothesis was partly based on in vitro evidence of increased staphylococcal binding to HeLa cells in the presence of estrogen [22]. The first epidemiological study showed an association between high circulating estrogen levels and staphylococcal nasal carriage among women [21]. In a cohort study among 694 healthy female volunteers in their third decade visiting a travel clinic, hormonal contraceptive (HC) users had an increased risk (OR 1.6, 95% CI 1.1–2.3) of *S*. *aureus* carriage at baseline and an increased risk (OR 3.2, 95% CI 1.4–7.3) of persistent *S*. *aureus* carriage after a median follow-up of 70 days, when compared to non-users [19]. However, it was impossible to separate the effect of progestin and estrogen as 96% used combination contraceptives. Another study based on nine HC users supports an association between oral hormonal contraceptives and nasal carriage [20]. Despite the high prevalence of *S*. *aureus* nasal carriage among adolescents, the association with HC use in women below 20 years has not been addressed.

In general, few host risk factors for *S*. *aureus* carriage have been investigated in adolescents, besides the link with atopic dermatitis [23] and low vitamin D as a predictor of methicillin resistant staphylococcus aureus (MRSA) nasal carriage [24]. Additional associations observed among adults, include higher *S*. *aureus* nasal carriage rates with obesity and type 2 diabetes [25], and lower rates in smokers [5].

The main aim of this study was to assess the association between overall use of HC and different types of HC, and *S*. *aureus* nasal and throat carriage in a population-based sample of healthy girls aged 17–21 years, and to test whether an association is independent of other known risk factors for *S*. *aureus* carriage.

## Methods

### Population and study design

**Fig 1:**
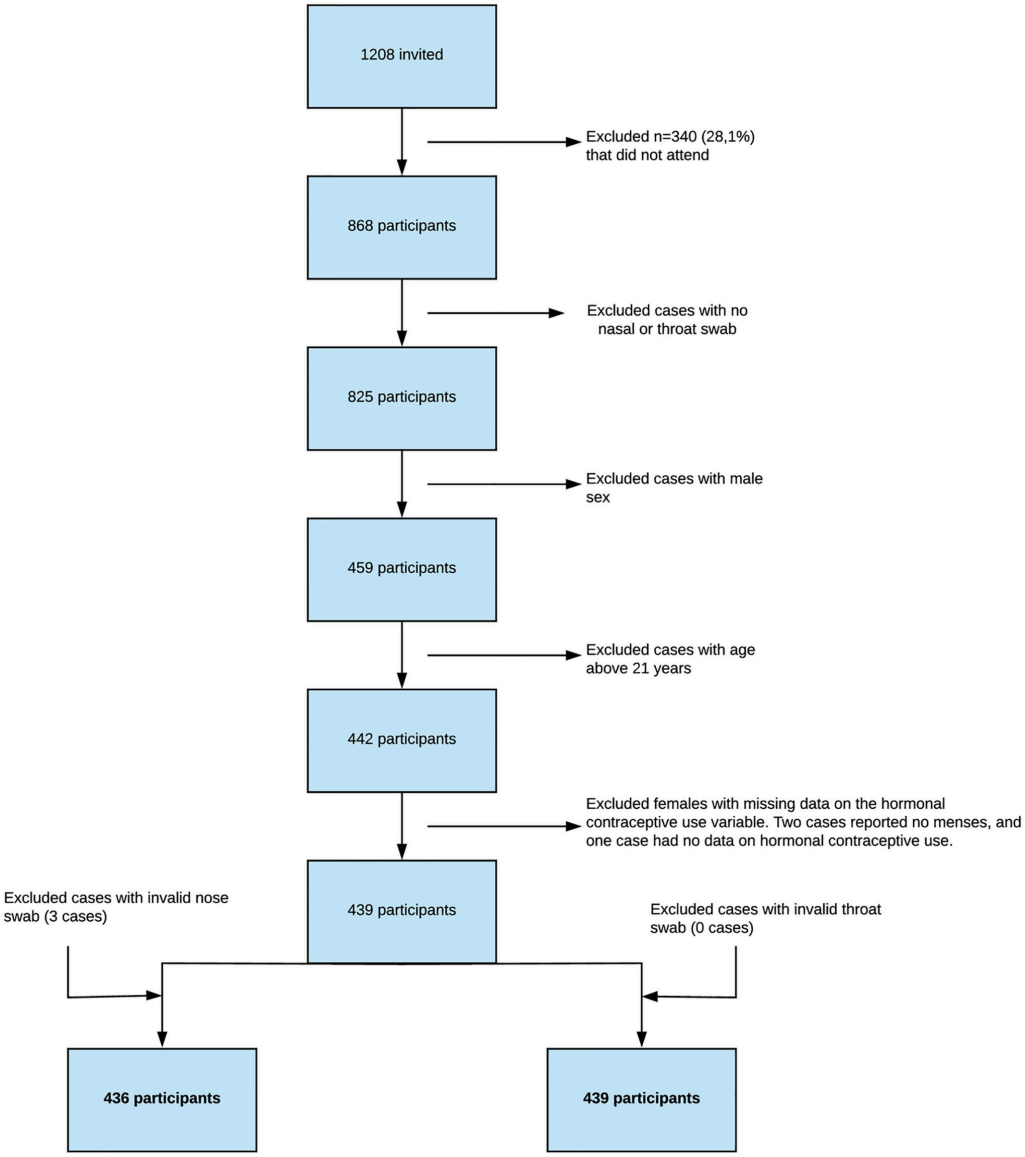
The study population. **The Tromsø Study, Fit Futures 2.** *68 throat cultures with no bacterial growth were recoded as valid swabs negative for *S*. *aureus*.

The Tromsø Study Fit Futures 1 and 2 (TFF1 and TFF2) comprise two waves of large population-based studies of lifestyle and health among upper-secondary school students in the Norwegian municipalities of Tromsø and Balsfjord [23, 26]. TFF2 was conducted in 2012–2013 and invited all third year students (n = 775), as well as TFF1 participants not attending school in 2012–2013 (n = 464). A total of 31 individuals (27 participants in TFF1 and 4 new students) could not be successfully contacted, and were not invited in TFF2. Among all students invited to TFF2 (n = 1208), 868 participated (71.9%). All males, participants with no or invalid nasal or throat swab, age exceeding 21 years, and females with missing data on HC use were excluded. The final study population included 436 and 439 women for the analysis of *S*. *aureus* nasal and throat carriage, respectively (Fig 1).

The participants had a half-day visit at The Clinical Research Unit, University Hospital of North Norway (UNN). A web-based questionnaire was used to collect data on lifestyle, health and disease. Nasal and throat swab cultures, clinical examinations, blood sampling, and interviews were performed by trained research nurses according to standardised procedures. Height and weight were measured on an electronic scale with lightweight clothing, and body mass index was calculated (kg/m^2^). Non-fasting blood samples were drawn from an antecubital vein. Methods for the assessment of EDTA-blood glycated hemoglobin (HbA1c) and serum 25-hydroxyvitamin D [25(OH)D] concentrations in TFF have been described previously [27].

### Assessment of *S*. *aureus* carriage

Nasal and throat swab samples were collected by research nurses. A NaCl (0.9%)-moistened sterile rayon-tipped swab rotated three times with a gentle pressure was used to sample both vestibule nasi. A second swab was used to sample both tonsillar regions with moderate pressure on the tonsillar surfaces. The swabs were immediately placed in transport medium (Amies Copan, Brescia, Italy) and stored at 4°C for 3–7 days (dx.doi.org/10.17504/protocols.io.2j5gcq6). The microbiological analysis was done by trained personnel at the Department of Medical Biology, Faculty of Health Sciences, UiT The Arctic University of Norway, Tromsø. First, the swabs were enriched in Bacto Staphylococcus medium broth (Difco laboratories, Sparks, MD, USA) and incubated for 18–24 hours at 37 °C. After enrichment, one drop of enrichment broth were streaked on blood agar (Oxoid, UK), CROMagar-plates for *S*. *aureus* detection (SmithMed AS/Microbiological media production and MRSA agar plates SmithMed AS/Microbiological media production, Department of Microbiology and Infection Control, UNN). In a pilot study among 10 adult volunteers, we demonstrated the validity of the method. One nasal swab sample from each nasal vestibule was taken and stored at 4°C for 9 days before enrichment and plating, gave the same culturing result as beginning the culturing with enrichment on the day of sampling. In order to increase statistical power and test for differences between agar plates, each nasal swab was streaked on blood agar and two different *S*. *aureus* growth media; the one used in the present study and chromId *S*. *aureus* agar (bioMérieux, France). The agar plates were incubated for 42–48 hours at 37°C. To retain high specificity, colony morphology was examined and the most dominating colony type on the SAID or MRSA plate was plated on blood agar and incubated for 20–24 hours at 37°C before confirmation as *S*. *aureus* by the Staphaurex plus agglutination test (Remel, USA). All confirmed *S*. *aureus* isolates were frozen at– 70 °C in glycerol-containing liquid media for molecular analysis (dx.doi.org/10.17504/protocols.io.2j6gcre). Growth of any bacterial colonies on any of the agar plates was registered as a valid culture. *S*. *aureus* carriage state was determined by nasal or throat swab positive for *S*. *aureus*. For throat cultures, all samples with no growth on neither plate, were recoded as valid cultures negative for *S*. *aureus* (n = 68). One MRSA isolate was confirmed from the nasal swabs. Two MRSA isolates from two different participants was confirmed from throat swabs. The verification of MRSA was done by detection of the thermostable nuclease of *S*. *aureus* and the *mecA* gene with the use of an in house real time PCR.

### Assessment of hormonal contraceptive use

Self-reported information on current contraceptive use was obtained by trained nurses asking female participants the interview questions: “If you have started menstruating; do you use any kind of contraceptives?” (yes/no), and “If you use any kind of contraceptives; what type?” (Tablets/Injections/Implants/Condom/Transdermal contraceptive patch/Vaginal contraceptive ring/Intrauterine device (IUD)/Other). Condom and other were defined as non-hormonal contraceptives. None used IUD as contraception, and the variable was excluded from further analysis.

HC users were asked about brand name of tablets, implants, transdermal contraceptive patch, or vaginal contraceptive ring, and all were shown photos of different brands sold in Norway to help correct reporting. In the analysis, HC types and brands were categorised into combination HC and progestin-only HC. The combination HCs were further divided into two groups according to the ethinylestradiol daily dosage (high and low). High dosage was defined as HC containing ≥ 30 μg ethinylestradiol. Low dosage was defined as contraceptives containing ≤ 20 μg ethinylestradiol. No HC brand contained between 20 and 30 μg ethinylestradiol.

### Statistical analysis

Univariable associations and differences between comparison groups were analyzed in contingency tables and by calculating means and standard deviations, using chi-square and t-tests to quantify the potential role of chance. Univariable and multivariable logistic regression were fitted to estimate odds ratios (ORs) and 95% confidence intervals (CIs) to describe the association between HC use and *S*. *aureus* nasal and throat carriage while adjusting for potential confounders. We used DAGitty 2.3 and Akaike Information Criterion (AIC) for model selection. Testing for potential interaction between explanatory variables was done by including the multiplicative terms of two predictor variables in the model and comparing models using AIC. We analyzed our data using SPSS version 23 and considered p-values < 0.05 as statistical evidence and 0.1 > p ≥ 0.05 as weak statistical evidence.

### Ethics

A declaration of consent was signed by each participant in TFF2. TFF2 was approved by the Regional Committee for Medical and Health Research Ethics (REK) and the Norwegian Data Protection Authority. The present study was approved by REK North, reference 2011/1710.

## Results

In this population of healthy female adolescents with mean age 18 years (range 17–21), the prevalence of *S*. *aureus* nasal carriage was 50.0% (218/436) while the prevalence of throat carriage was 49.7% (263/439). Simultaneous nasal and throat carriage were found in 178 participants. Table 1 shows selected population characteristics by *S*. *aureus* nasal and throat carriage types. *S*. *aureus* nasal carriage was positively associated with self-reported doctor diagnosed atopic eczema, low recreational physical activity, daily snuff use, and use of alcohol (all p < 0.05). *S*. *aureus* throat carriage was associated with low recreational physical activity and use of alcohol, while use of antibiotics the past three months was negatively associated with throat carriage (all p < 0.05).

**Table 1:** Characteristics of the study population by *S*. *aureus* nasal and throat carriage. The Tromsø Study Fit Futures 2.

	Nasal carriage (n = 436)				Throat carriage (n = 439)			
	Non-carrier (n = 218)	Carrier (n = 218)	*P*^a^	OR (95%CI)^b^	Non-carrier (n = 176)	Carrier (n = 263)	*P*^a^	OR (95% CI)^b^
**Age**, years (mean, SD)	18.2 (0.7)	18.3 (0.7)	.37	1.13 (0.86–1.48)	18.3 (0.7)	18.3 (0.7)	.65	0.86 (0.63–1.18)
**BMI**, kg/m^2^ (mean, SD)	23.2 (4.3)	23.1 (4.3)	.74	0.99 (0.95–1.04)	23.3 (4.4)	23.0 (4.1)	.53	0.97 (0.93–1.02)
**BMI-category**			.66				.36	
< 18.5 kg/m2	12 (57.1)	9 (42.9)		0.7 (0.29–1.70)	10 (47.6)	11 (52.4)		0.48 (0.19–1.24)
18.5-<25 kg/m2	154 (48.3)	165 (51.7)		1.0 (ref)	121 (37.6)	201 (62.4)		1.0 (ref)
25-<30 kg/m2	35 (55.6)	28 (44.4)		0.75 (0.43–1.29)	29 (46.0)	34 (54.0)		0.74 (0.39–1.42)
30 + kg/m2	17 (51.5)	16 (48.5)		0.88 (0.43–1.80)	16 (48.5)	17 (51.5)		0.42 (0.20–0.90)
**HbA1c** (mean, SD)	5.4 (0.4)	5.3(0.3)	.05	0.49 (0.24–1.02)	5.4 (0.4)	5.3 (0.3)	.03	0.64 (0.26–1.58)
**Vitamin D** (mean, SD)	49.5 (22.9)	51.1 (22.9)	.15	1.01 (0.99–1.02)	51.3 (23.7)	50.9 (22.4)	.83	1.01 (0.99–1.01)
**Vitamin D group**^c^			.09				.64	
Deficiency	25 (53.2)	22 (46.8)		0.77 (0.40–1.45)	18 (37.5)	30 (62.5)		0.99 (0.47–2.11)
Insufficiency	95 (58.3)	68 (41.7)		0.62 (0.40–0.95)	73 (44.5)	91 (55.5)		0.80 (0.49–1.3)
Normal	87 (46.5)	100 (53.5)		1.0 (ref)	77 (41.0)	111 (59.0)		1.0 (ref)
**Smoking**			.17				.71	
Yes	50 (56.8)	38 (43.2)		0.7 (0.44–1.12)	37 (42.0)	51 (58.0)		0.82 (0.47–1.42)
No	164 (48.0)	178 (52.0)		1.0 (ref)	135 (39.1)	210 (60.9)		1.0 (ref)
**Daily snuff use**			.03				.11	
Yes	47 (40.9)	68 (59.1)		1.63 (1.06–2.51)	38 (33.0)	77 (67.0)		1.50 (0.88–2.56)
No	168 (53.0)	149 (47.0)		1.0 (ref)	135 (42.2)	185 (57.8)		1.0 (ref)
**Alcohol use**			.03				.009	
> 4 times/month	11 (57.9)	8 (42.1)		2.09 (0.62–7.05)	8 (42.1)	11 (57.9)		2.75 (0.77–9.86)
2–4 times/month	111 (48.9)	116 (51.1)		3.01 (1.29–6.99)	88 (38.8)	139 (61.2)		4.63 (1.95–11.04)
≤ 1 time/month	70 (45.2)	85 (54.8)		3.49 (1.47–8.28)	56 (35.4)	102 (64.6)		3.83 (1.59–9.22)
Never	23 (74.2)	8 (25.8)		1.0 (ref)	21 (67.7)	10 (32.3)		1.0 (ref)
**Physical activity**^d^			.02				.05	
Low level	21 (34.4)	40 (65.6)		2.35 (1.29–4.30)	20 (32.3)	42 (67.7)		1.83 (0.91–3.71)
Medium level	100 (55.2)	81 (44.8)		1.0 (ref)	84 (46.4)	97 (53.6)		1.0 (ref)
High level	92 (48.9)	96 (51.1)		1.29 (0.86–1.94)	68 (35.8)	122 (64.2)		1.87 (1.14–3.07)
**Atopic eczema**			.007				.37	
Yes	19 (32.8)	39 (67.2)		2.28 (1.27–4.09)	20 (33.9)	39 (66.1)		1.71 (0.82–3.56)
No	199 (52.6)	179 (47.4)		1.0 (ref)	156 (41.1)	224 (58.9)		1.0 (ref)
**Antibiotic use past 3 months**			.36				.01	
Yes	39 (55.7)	31 (44.3)		0.76 (0.45–1.27)	38 (54.3)	32 (45.7)		0.54 (0.30–0.98)
No	179 (48.9)	187 (51.1)		1.0	138 (37.4)	231 (62.6)		1.0 (ref)

Values are number of subjects (%) if not otherwise stated.

BMI = body mass index; SD = standard deviation; HbA1c, glycated haemoglobin.

^a^ Chi-square test for categorical and t-tests for continuous variables.

^b^ Univariable logistic regression analysis. OR = Odd ratio CI = 95% confidence intervals

^c^ Serum 25-hydroxyvitamin D: Deficiency = < 25 nmol/l; Insufficiency = 25–50 nmol/l; Normal = >50 nmol/l.

^d^ Recreational physical activity: Low level = reading, watching TV, or other sedentary activity; Medium level = walking, cycling, or other forms of exercise at least 4 hours a week; High level = participation in recreational sports, heavy outdoor activities with minimum duration of 4 hours a week, or participation in heavy training or sports competitions regularly several times a week.

A higher proportion of women using combination HC were nasal carriers compared to non-users, and there was a positive association with higher oestrogen dosage. Progestin-only contraceptives were negatively associated with nasal carriage (p < 0.001) (Table 2). There was no evidence for differences in prevalence of throat carriage across categories of HC use.

**Table 2:** Prevalence of *S*. *aureus* nasal and throat carriage by group of hormonal contraceptive use. The Tromsø Study Fit Futures 2.

	Nasal carriage (n = 436)			Throat carriage (n = 439)		
	Non-carrier (n = 218) n (%)	Carrier (n = 218) n (%)	P-value^a^	Non-carrier (n = 176) n (%)	Carrier (n = 263) n (%)	P-value^a^
Non-user	113 (58.2)	81 (41.8)	<.001	84 (42.4)	114 (57.6)	.460
Combination with Low estrogen^b^	36 (44.4)	45 (55.6)		27 (34.6)	51 (65.4)	
Combination with High estrogen ^c^	44 (35.8)	79 (64.2)		47 (37.6)	78 (62.4)	
Progestin-only^d^	25 (65.8)	13 (34.2)		18 (47.4)	20 (52.6)	

^a^ Chi-square test

^b^ Combination contraceptive with ethinyl estradiol dosage less than or equal to 20μg. Mercilon, Yasminelle, Loette 28, Nuvaring.

^c^ Combination contraceptive with ethinyl estradiol dosage greater than or equal to 30μg. Marvelon, Yasmin, Microgynon, Oralcon, Diane, Synfase, Evra

^d^ Progestin-only contraceptives. Cerazette, Nexplanon, Depo-provera.

In a multivariable logistic regression model, users of high dosage estrogen HC had a 2.4 fold higher odds of *S*. *aureus* nasal carriage as compared with non-users (OR = 2.44; 95%CI = 1.39–4.28, adjusted for age, BMI, smoking, snuff- and alcohol use, recreational physical activity, HbA1c and 25-OH-vitamin D levels, atopic eczema and the use of antibiotics in the past three months) (Table 3). Users of low dosage estrogen HC had an adjusted OR of 2.14 compared to non-users (95%CI = 1.17–3.91). Users of progestin-only contraceptives had an adjusted OR of 0.29 (95%CI = 0.12–0.67) compared to users of combination contraceptives (results not presented in tables). In the same model, higher odds of *S*. *aureus* nasal carriage was also observed for atopic eczema (OR = 2.50; 95%CI = 1.28–4.89), low physical activity (OR = 2.12; 95%CI = 1.06–4.23), use of alcohol once a month or less (OR = 3.81; 95%CI = 1.42–10.23), and use of alcohol 2–4 times a month (OR = 3.48; 95%CI = 1.32–9.21). Weak statistical evidence was found for a negative association with increasing HbA1c (OR = 0.52; 95%CI = 0.23–1.20).

**Table 3:** Associations between hormonal contraceptive use and *S*. *aureus* nasal and throat carriage. Odd ratios (OR) and 95% confidence intervals (95%CI) from multivariable logistic regression analysis.^a^ The Tromsø Study Fit Futures 2.

	Nasal carriage (n = 436)	Throat carriage (n = 439)
	OR (95%CI)	OR (95%CI)
**Hormonal contraceptive use**		
Non-user	1.0 (ref)	1.0 (ref)
Progestin-only^b^	0.63 (0.27–1.45)	0.90 (0.37–2.19)
Combination with low estrogen^c^	2.14 (1.17–3.91)	1.79 (0.89–3.60)
Combination with high estrogen^d^	2.44 (1.39–4.28)	2.06 (0.96–4.44)
**Age continuous**	1.15 (0.84–1.58)	0.93 (0.65–1.32)
**BMI continuous**	0.99 (0.94–1.05)	0.96 (0.90–1.02)
**HbA1c continuous**	0.52 (0.23–1.20)	0.72 (0.26–1.96)
**Vitamin D continuous**	1.00 (0.99–1.01)	0.99 (0.98–1.00)
**Atopic eczema**		
Yes	2.50 (1.28–4.89)	1.49 (0.68–3.31)
No	1.0 (ref)	1.0 (ref)
**Smoking**		
Yes	0.53 (0.30–0.95)	0.75 (0.39–1.47)
No	1.0 (ref)	1.0 (ref)
**Daily snuff use**		
Yes	1.39 (0.82–2.37)	1.55 (0.82–2.94)
No	1.0 (ref)	1.0 (ref)
**Alcohol use**		
More than 4 times a month	2.12 (0.50–8.94)	1.98 (0.45–8.72)
2–4 times a month	3.48 (1.32–9.21)	3.85 (1.49–9.95)
Once a month or less	3.81 (1.42–10.23)	3.39 (1.29–8.86)
Never	1.0 (ref)	1.0 (ref)
**Recreational physical activity**^e^		
Low level	2.12 (1.06–4.23)	1.35 (0.62–2.93)
Medium level	1.0 (ref)	1.0 (ref)
High level	1.30 (0.80–2.11)	1.83 (1.04–3.21)
**Antibiotic use past 3 months**		
Yes	0.88 (0.49–1.57)	0.63 (0.32–1.24)
No	1.0 (ref)	1.0 (ref)

BMI = body mass index; HbA1c, glycated haemoglobin.

^a^ All variables in the table are mutually adjusted for

^b^ Progestin-only = Cerazette, Nexplanon, Depo-provera.

^c^ Combination contraceptive with ethinyl estradiol dosage less than or equal to 20μg. Mercilon, Yasminelle, Loette 28, Nuvaring.

^d^ Combination contraceptive with ethinyl estradiol dosage greater than or equal to 30μg. Marvelon, Yasmin, Microgynon, Oralcon, Diane, Synfase, Evra

^e^ Recreational physical activity: Low level = reading, watching TV, or other sedentary activity; Medium level = Walking, cycling, or other forms of exercise at least 4 hours a week; High level = Participation in recreational sports, heavy outdoor activities with minimum duration of 4 hours a week, or participation in heavy training or sports competitions regularly several times a week.

Multivariable logistic regression analysis of *S*. *aureus* throat carriage showed a trend towards modestly higher odds for participants using high (OR = 2.06; 95%CI = 0.96–4.44), and low dosage (OR = 1.79; 95%CI = 0.89–3.60), combination HC versus non-users, but this finding could also be due to chance (Table 3). There was a statistically significant higher odds of *S*. *aureus* throat carriage for the use of alcohol once a month or less (OR = 3.39; 95%CI = 1.29–8.86), for alcohol use 2–4 times a month (OR = 3.85; 95%-CI = 1.49–9.95), and for high level physical activity (OR = 1.83; 95%CI = 1.04–3.21).

Test for interaction was performed by including multiplicative terms of two and two predictors in the logistic regression model. In the analysis for throat carriage, four interactions were detected. There was an interaction between atopic eczema and HbA1c (p = 0.01), between antibiotic use and alcohol use (p = 0.02), between age and smoking status (p = 0.04) and between smoking and HbA1c (p = 0.05). In the analysis for nasal carriage, there was an interaction between physical activity and HbA1c (p<0.00) and between use of antibiotics and smoking status (p = 0.04). None of the interactions affected the main result and where therefore not included in the final analysis. We performed sensitivity analysis to check the robustness of our results. The results presented in tables were generated from multivariable logistic regression analysis in which observations with missing values were excluded. A multiple imputation analysis was used to evaluate the effect of differently handled data analysis, but the estimates were not significantly changed.

## Discussion

This is the first report on the association between use of HC and *S*. *aureus* carriage based on a representative sample of healthy women aged 17–21 years. We have demonstrated a strong association between use of combination HC and nasal carriage, where the association is strengthen with higher dosage of estrogen. Women using combination HC had more than doubled odds for nasal carriage compared with non-users, suggesting that exogenous estrogen is a major predictor of *S*. *aureus* nasal carriage. Among users of combination HC containing both progestin and estrogen, the *S*. *aureus* nasal carriage prevalence was 64.2% (79/123) in the high-estrogen group and 55.6% (45/81) in the low-estrogen group. This may suggest a dose-response relationship. In contrast, users of progestin-only HC had an OR of 0.29 for nasal carriage compared to women using combination therapy. This substantial difference in risk between the HC-user groups suggests that estrogen and progestin have opposite immune-modulatory effects on *S*. *aureus* colonization, and that the risk associated with exogenous estrogen alone is considerably higher than that of combination HC.

Our findings are supported by three epidemiological studies in adults, one on endogenous estrogen [21] and two on HC use [19–21]. Zanger *et al* did the first study of HC use in a larger population of 694 women in Germany [19]. The doubled odds of *S*. *aureus* nasal carriage in our study lies between their observed 60% higher risk of *S*. *aureus* nasal carriage at baseline and tripled risk of being persistent carrier after two months follow-up. These authors took two nasal swabs at each time point, and defined only those with both samples positive for *S*. *aureus* after direct culturing as carriers, while in the TTF2 we used one nasal swab cultured with enrichment broth. This could explain some of the difference in risk estimates between the studies. Also, there were differences in age, type of source population (visitors to travel clinic versus students in upper-secondary school) and access to information on possible confounding factors. The other study on HC use and *S*. *aureus* has limited validity as the sample size was small [20]. Our study provides the first evidence for differences in the effect of progestin-only and combination HC on *S*. *aureus* colonization, as hypothesised by Zanger’s group, who were unable to explore this due to the low prevalence of progestin-only users [19].

Nasal carriage of *S*. *aureus* is generally considered as a subclinical inflammatory process due to suppression of the innate immune system [28]. Immune responses vary with gender and the reproductive phase, suggesting that factors associated with reproduction regulate immune response [29]. There have been conflicting results on the association between HC use and immunoglobulin production [30–32]. One study showed decreased immunoglobulin levels in HC users [32]. A recent study demonstrated an association between higher Toll-like receptor 9 (TLR9) transcription levels and non-carrier status of *S*. *aureus* [33]. This study also showed that both the association with TLR9 genotype and transcription level were modified by sex, suggesting a role of reproductive hormones in *S*. *aureus* immunity. This may be underlying biological mechanisms that can explain why users of combination HC are more likely to be nasal carriers of *S*. *aureus*.

Though not statistically significant, our analysis shows a lower risk of *S*. *aureus* nasal carriage in progestin-only users compared to non-users, contrasting the higher risk among users of combination HC. These differences point to direct exogenous hormonal effects, and that the observed associations are not due to environmental factors such as differences in human contact between non-user group and user group. Nevertheless, we cannot rule out that unknown risk factors may account for some of the observed associations.

The study demonstrate a high overall prevalence in *S*. *aureus* carriage with a prevalence of nasal carriage of 50% (218/436) and a prevalence for throat carriage of 60% (263/439). In a previous validation study (unpublished), the prevalence of enriched samples were 70% for throat samples and 49% for nasal samples. When using direct cultivation of the swabs we detected a prevalence of 36% for throat samples and 36% for nasal samples. The overall high prevalence in our study is the result of the method used and must be taken in to consideration when comparing to other studies.

The main strengths in our study include a population-based design with high attendance and thereby reduced risk of selection bias. Exogenous hormone exposure was assessed in photo-assisted interviews by trained nurses to reduce the risk of information bias. Thus, we may assume that the TFF2 data are representative for Norwegian adolescents and youth populations from similar modern societies. As TFF2 includes detailed information on lifestyle and health, we were able to adjust for possible confounding. DAGitty 2.3 and Akaike Information Criterion (AIC) were used for selecting the optimal regression model.

We included both smoking status and snuff use as covariates in the model as smoking has been consistently associated with lower *S*. *aureus* nasal carriage in adults [1, 5–6]. Considering that this is a healthy, young population the value of adjusting for HbA1c can be debated. Very few subjects reported no alcohol use or use more than 4 times per month. The association between alcohol use and carriage should therefore be interpreted with caution.

A weakness in our study is that only one sample from throat and nose was taken from each participant. This renders it impossible to distinguish between persistent and intermittent carriers and may represent a detection bias of unknown effect. Due to the enrichment step before plating of the nasal and throat swab cultures, quantification of *S*. *aureus* growth was irrelevant. Data on duration of HC use and thereby cumulative exogenous hormone exposure was unavailable. Thus, we were unable to test for these possible dose-response relationships.

We did not find the same association between HC use and *S*. *aureus* carriage based on throat samples as with nasal samples. This may represent a true difference in the host-microbe relationship between throat and nose. However, there are some methodological concerns relating to the throat swabs in our study. A possible source of bias can be the more complicated sampling method for throat swabs with lower compliance due to participant discomfort. Furthermore, we chose to include throat samples without any bacterial growth on the agar plates in our analysis. The enrichment broth promotes growth of staphylococci, while other members of upper respiratory microbiota are inhibited by the relatively high concentration of sodium chloride. Therefore, agar plates with no bacterial growth were included in the study as valid negative throat samples. The pilot study showed that nasal swab samples cultured within 9 days after sampling gave the same culturing results on both control agar and *S*. *aureus* agar plates as culturing on the day of sampling. We did not include throat swab samples in the pilot study. However, we assume that for throat swabs *S*. *aureus* culture has similar validity as nasal swabs, while growth on control agar has lower validity. All the potential sources of bias mentioned are sources of non-differential bias, as there is no obvious link to HC use.

In summary, we report novel evidence for an association between use of HC and risk of *S*. *aureus* nasal carriage in female adolescents. Furthermore we observe that progestin-estrogen combination users have higher risk while progestin-only users have lower risk of nasal carriage compared to non-users. Our data support that exogenous estrogen is a major risk factor with potentially large impact on the *S*. *aureus* burden in the youth population. Our study has a biological foundation [33], demonstrates a dose response relationship, and the results are supported by data from another study including a different group of participants [19]. This study, together with existing knowledge, provides evidence for a causal association between exogenous estrogen exposure from hormonal contraceptives and nasal carriage of *S*. *aureus*. An experimental study design is needed to establish the role of exogenous sex steroids in *S*. *aureus* colonization in women.

## Supporting information

S1 Table Table of variables. (DOCX)

S1 Fig Figure of DAGitty models and corresponding AICs. (PDF)

S1 Text General questionnaire. Questionnaire from TFF2 in original language.
(PDF)

S2 Text Interview. Interview from TFF2 in original language.
(DOC)

S3 Text Metadata. Questionnaire and Interview for TFF2 in English.
(XLS)
