# Supplementary material for: Hormonal contraceptive use and Staphylococcus aureus nasal and throat carriage in a Norwegian youth population
Source: PLoS One. 2019 Jul 5;14(7):e0218511. doi: 10.1371/journal.pone.0218511 (PMC6611591; doi:10.1371/journal.pone.0218511)
Supplement: S1 Table — (DOCX) [file pone.0218511.s001.docx]

# Table of variables

| **Variable** | **Type** |
| --- | --- |
| Sex | Categorical |
| Age | Continuous |
| Participation consent | Categorical |
| Attendance date | Date format |
| Healthy today | Categorical |
| Fever today | Categorical |
| Common cold today | Categorical |
| Infection today | Categorical |
| Infection today type | Categorical |
| Chronic disease | Categorical |
| Diagnosis chronic disease | Text |
| ICD10 Chronic disease(calculated) | Text |
| Age diagnosis chronic disease | Continuous |
| Diagnosis chronic disease 2 | Text |
| ICD10 Chronic disease 2(calculated) | Text |
| Age diagnosis chronic disease2 | Continuous |
| Diagnosis chronic disease3 | Text |
| ICD10 Chronic disease3(calculated) | Text |
| Age diagnosis chronic disease3 | Continuous |
| Diagnosis chronic disease4 | Text |
| ICD10 Chronic disease4(calculated) | Text |
| Age diagnosis chronic disease4 | Continuous |
| Diagnosis chronic disease5 | Text |
| ICD10 Chronic disease5(calculated) | Text |
| Age diagnosis chronic disease5 | Continuous |
| Chronic disease other | Text |
| Age chronic disease other | Continuous |
| Antibiotics the last 3 months | Categorical |
| Antibiotics last 3 months brand | Text |
| Antibiotics the last 3 months ATC-code | Text |
| Weeks since last taken antibiotic | Continuous |
| Antibiotics last 3 months brand2 | Text |
| Antibiotics the last 3 months ATC-code2 | Text |
| Weeks since last taken antibiotic2 | Continuous |
| Antibiotics last 3 months brand3 | Text |
| Antibiotics the last 3 months ATC-code3 | Text |
| Weeks since last taken antibiotic3 | Continuous |
| Antibiotics last 24 h | Categorical |
| Antibiotics last 24 h brand | Text |
| Antibiotics the last 24h ATC-code | Text |
| Antibiotics last 24 h brand2 | Text |
| Antibiotics the last 24h ATC-code2 | Text |
| Antibiotics last 24 h brand3 | Text |
| Antibiotics the last 24h ATC-code3 | Text |
| Menses | Categorical |
| Menses, regularity | Categorical |
| Menses, cycle length | Continuous |
| Last menses, certainty of start date | Categorical |
| Date of last menstruation | Date format |
| Contraceptives | Categorical |
| Contraceptives type | Categorical |
| Oral contraceptives name | Text |
| Injected contraceptives name | Text |
| Subdermal contraceptives name | Text |
| Contraceptive skin patch name | Text |
| Vaginal contraceptives name | Text |
| IUD name | Text |
| Oral contraceptives ATC-code | Text |
| Injected contraceptives ATC-code | Text |
| Subdermal contraceptives ATC-code | Text |
| Contraceptive skin patch ATC-code | Text |
| Vaginal contraceptives ATC-code | Text |
| IUD ATC-code | Text |
| Chance of pregnancy | Categorical |
| Pregnancy test consent | Categorical |
| Pregnancy test result | Categorical |
| Eczema today | Categorical |
| Antibiotics local | Categorical |
| Antibiotics local brand | Categorical |
| Antibiotics local ATC | Categorical |
| Antibiotics local 2 | Categorical |
| Antibiotics local brand 2 | Categorical |
| Antibiotics local ATC 2 | Categorical |
| UV-treatment last 14 days | Categorical |
| Height | Continuous |
| Weight | Continuous |
| Waist, 1. Measurement | Continuous |
| Hip, 1. Measurement | Continuous |
| Waist, 2. Measurement | Continuous |
| Hip, 2. Measurement | Continuous |
| Growth on control agar, nasal swab | Categorical |
| Growth on staphylococcal selective agar | Categorical |
| Coagulase test, nasal swab | Categorical |
| Staphylococcus aureus (coagulase positive), with bacterial growth | Categorical |
| Comments, lab staphylococcus aureus | Text |
| Staphylococcus culture date | Date format |
| BMI (calculated) | Categorical |
| Atopic eczema | Categorical |
| Smoking | Categorical |
| Smoking, start age | Categorical |
| Cigarettes per week | Categorical |
| Cigarettes per day | Categorical |
| Snuff | Categorical |
| Snuff, start age | Categorical |
| Snuff, portion per week | Categorical |
| Snuff portion per day | Categorical |
| Alcohol frequency | Categorical |
| Alcohol units | Categorical |
| Alcohol frequency of 6 units or more | Categorical |
| Physical activity | Categorical |
| Physical activity outside of school | Categorical |
| Physical activity, frequency | Categorical |
| Physical activity, intensity | Categorical |
| Acne, lifetime | Categorical |
| Acne, severity | Categorical |
| Acne, been seeing a doctor | Categorical |
| Acne, local treatment | Categorical |
| Acne, antibiotic therapy | Categorical |
| Acne, roaccutan treatment | Categorical |
| Dry skin | Categorical |
| Dry skin lotion | Categorical |
| Itchy rash | Categorical |
| Itchy rash duration | Categorical |
| Itchy rash head | Categorical |
| Itchy rash face | Categorical |
| Itchy rash ear | Categorical |
| Itchy rash neck | Categorical |
| Itchy rash wrist | Categorical |
| Itchy rash hands | Categorical |
| Itchy rash buttocks | Categorical |
| Itchy rash inner thigh | Categorical |
| Itchy rash truncus | Categorical |
| Itchy rash armpits | Categorical |
| Itchy rash groin | Categorical |
| Itchy rash outer extremities | Categorical |
| Itchy rash feet | Categorical |
| Itchy rash inner elbow/knee | Categorical |
| Itchy rash other | Categorical |
| Itchy rash debut age | Continuous |
| Itchy rash January | Categorical |
| Itchy rash February | Categorical |
| Itchy rash March | Categorical |
| Itchy rash April | Categorical |
| Itchy rash May | Categorical |
| Itchy rash June | Categorical |
| Itchy rash July | Categorical |
| Itchy rash August | Categorical |
| Itchy rash September | Categorical |
| Itchy rash October | Categorical |
| Itchy rash November | Categorical |
| Itchy rash December | Categorical |
| Itchy rash Cleared | Categorical |
| Itchy rash awake at night | Categorical |
| Itchy rash week | Categorical |
| Itchy rash week school | Categorical |
| Itchy rash week pain | Categorical |
| Itchy rash week sad | Categorical |
| Itchy rash week friends | Categorical |
| Itchy rash week clothes | Categorical |
| Itchy rash going out | Categorical |
| Itchy rash swimming | Categorical |
| Itchy rash week holiday | Categorical |
| Itchy rash week teasing | Categorical |
| Itchy rash week sleep | Categorical |
| Itchy rash week problems | Categorical |
| Eczema past year | Categorical |
| Eczema past year duration | Categorical |
| Eczema past year cortisone | Categorical |
| Eczema school/work | Categorical |
| Eczema leisure activities | Categorical |
| Eczema worries | Text |
| Hormonal Contraceptives (calculated) | Categorical |
| 25-OH vitamin D | Continuous |
| HbA1c | Continuous |
| Staph. Eczema culture positive | Categorical |
| Staph skin culture positive | Categorical |
| Staph nasal culture positive | Categorical |
| Staph nasal all | Categorical |
| Staph throat all | Categorical |
