# Supplementary material for: Hormonal contraceptive use and Staphylococcus aureus nasal and throat carriage in a Norwegian youth population
Source: PLoS One. 2019 Jul 5;14(7):e0218511. doi: 10.1371/journal.pone.0218511 (PMC6611591; doi:10.1371/journal.pone.0218511)
Supplement: S1 Fig — (PDF) [file pone.0218511.s002.pdf]

Model selection

Final model

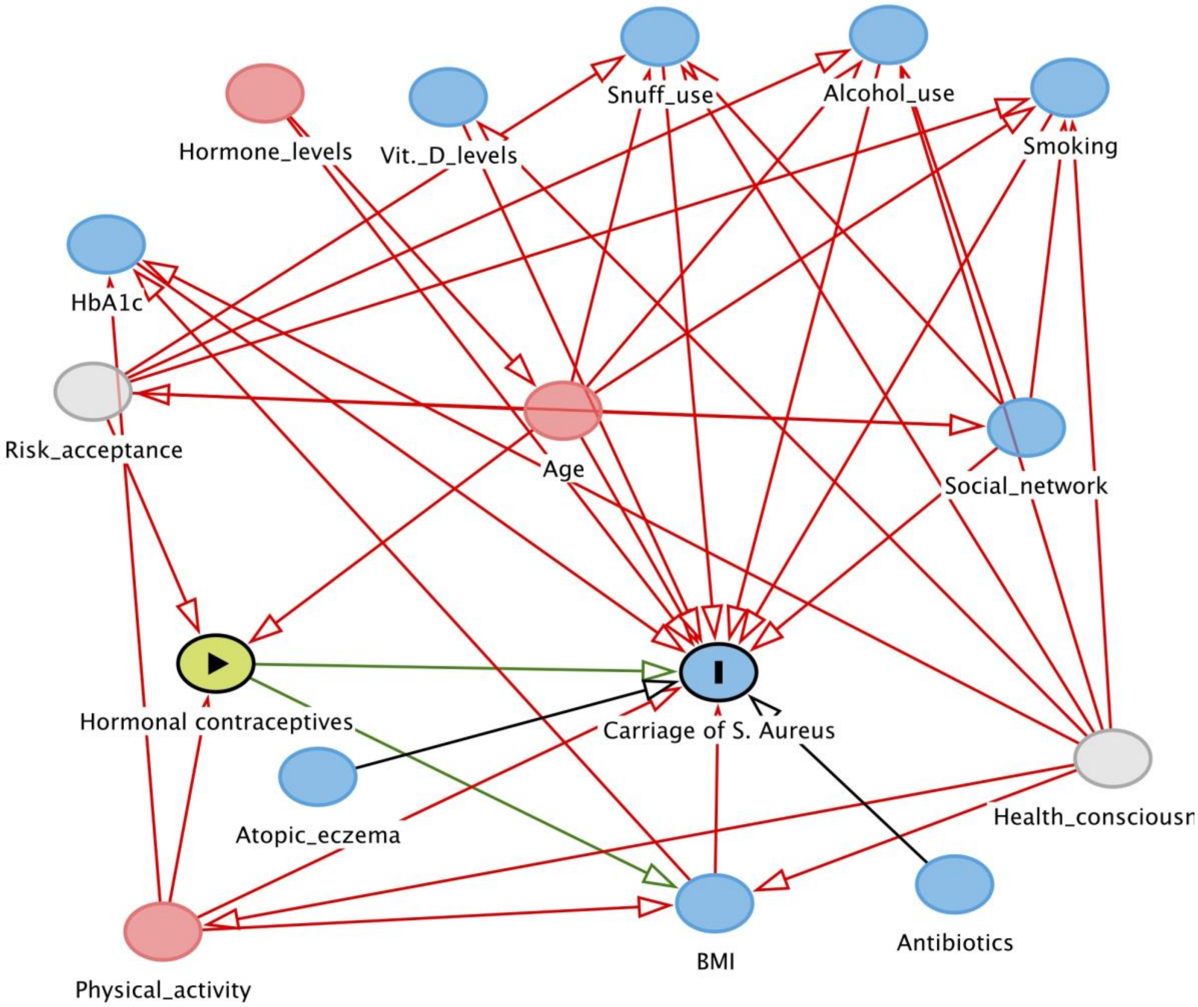

Minimal sufficient adjustment sets for estimating the direct effect of HC on carriage of S. aureus:
Age, alcohol use, BMI, HbA1c, physical activity, smoking, snuff use, social network, vit. D levels

Directed Acyclic Graphs (www.dagitty.net) of final model

Likelihood Ratio Tests

Effect	Model Fitting Criteria			Likelihood Ratio Tests		
	AIC of Reduced Model	BIC of Reduced Model	-2 Log Likelihood of Reduced Model	Chi-Square	df	Sig.
Intercept	511,646	574,898	479,646[a]	,000	0	.
AGE_FF2	510,426	569,725	480,426	,780	1	,377
bmi_ff2	509,710	569,009	479,710	,064	1	,800
S_25_VITD_FF2	509,655	568,954	479,655	,009	1	,925
S_HBA1C_FF2	512,321	571,620	482,321	2,675	1	,102
SMOKE_GROUPS	514,214	573,513	484,214	4,568	1	,033
SNUFF_GROUPS	511,314	570,613	481,314	1,668	1	,197
ALCOHOL_GROUPS	514,670	566,062	488,670	9,024	3	,029
PHYS_ACT_GROUPS	512,482	567,828	484,482	4,836	2	,089
ANTIBIOTICS_GROUPS	509,826	569,125	479,826	,180	1	,671
ATOPIC_ECZEMA	517,317	576,616	487,317	7,671	1	,006
HORMONAL_CONTRACEPTIVES2	524,862	580,208	496,862	17,216	2	,000

The chi-square statistic is the difference in -2 log-likelihoods between the final model and a reduced model. The reduced model is formed by omitting an effect from the final model. The null hypothesis is that all parameters of that effect are 0.

a. This reduced model is equivalent to the final model because omitting the effect does not increase the degrees of freedom.

Akaike Information Criteria (AIC) of final model for nasal carriage

Likelihood Ratio Tests

Effect	Model Fitting Criteria			Likelihood Ratio Tests		
	AIC of Reduced Model	BIC of Reduced Model	-2 Log Likelihood of Reduced Model	Chi-Square	df	Sig.
Intercept	528,996	592,372	496,996[a]	,000	0	.
S_25_VITD_FF2	529,679	589,094	499,679	2,683	1	,101
S_HBA1C_FF2	531,088	590,503	501,088	4,092	1	,043
bmi_ff2	527,612	587,027	497,612	,616	1	,433
AGE_FF2	527,137	586,552	497,137	,141	1	,707
HORMONAL_CONTRACEPTIVE_GROUP2	527,022	582,476	499,022	2,026	2	,363
ATOPIC_ECZEMA	527,254	586,669	497,254	,257	1	,612
ANTIBIOTICS	531,893	591,308	501,893	4,896	1	,027
SMOKE_GROUPS	527,475	586,891	497,475	,479	1	,489
SNUFF_GROUPS	528,160	587,575	498,160	1,164	1	,281
ALCOHOL_GROUPS	532,211	583,705	506,211	9,215	3	,027
PHYS_ACT_GROUPS	528,773	584,227	500,773	3,777	2	,151

The chi-square statistic is the difference in -2 log-likelihoods between the final model and a reduced model. The reduced model is formed by omitting an effect from the final model. The null hypothesis is that all parameters of that effect are 0.

a. This reduced model is equivalent to the final model because omitting the effect does not increase the degrees of freedom.

Akaike Information Criteria (AIC) of final model for throat carriage
