# Supplementary material for: Hormonal contraceptive use and Staphylococcus aureus nasal and throat carriage in a Norwegian youth population
Source: PLoS One. 2019 Jul 5;14(7):e0218511. doi: 10.1371/journal.pone.0218511 (PMC6611591; doi:10.1371/journal.pone.0218511)
Supplement: S1 Text — Questionnaire from TFF2 in original language. (PDF) [file pone.0218511.s003.pdf]

FF2 Generelt spørreskjema - UKE 1

Vi ønsker å vite mer om livsstil og helse.

Bruk den tiden du trenger til å svare så presist du kan.

Alle svarene dine blir behandlet med taushetsplikt.

Bruk "neste >>" og "<< tilbake" - knappene i skjema for å bla deg fremover og bakover.

Lykke til og tusen takk for hjelpen!

DEG OG DIN FAMILIE

1) Er du:

- Jente
- Gutt

2) Hvem bor du sammen med nå? (sett ett eller flere kryss)

- ☐ Mor
- ☐ Far
- ☐ 1-2 søsken
- ☐ 3 eller flere søsken
- ☐ Mors nye mann/samboer
- ☐ Fars nye kone/samboer
- ☐ Fosterforeldre
- ☐ Adoptivforeldre
- ☐ Besteforeldre
- ☐ Venner
- ☐ Alene/på hybel
- ☐ Institusjon
- ☐ Samboer/gift
- ☐ Annet

Denne informasjonen vises kun i forhåndsvisningen

Følgende betingelser må være oppfylt for at spørsmålet skal vises for respondenten:

Dersom spørsmålet “Hvem bor du sammen med nå? (sett ett eller flere kryss)” inneholder noen av disse alternativene

- “Institusjon”
- “Alene/på hybel”
- “Venner”

3) Hvor lenge er det siden du flyttet hjemmefra?

- Mindre enn 6 måneder
- 6 - 11 måneder
- 1 - 2 år
- Mer enn 2 år

4) Er moren din i arbeid? (sett ett eller flere kryss)

- ☐ Ja, heltid
- ☐ Ja, deltid
- ☐ Arbeidsledig
- ☐ Uførerygdet
- ☐ Hjemmeværende
- ☐ Går på skole, kurs, e.l.
- ☐ Pensjonist
- ☐ Mor er død
- ☐ Vet ikke
- ☐ Annet

5) Er faren din i arbeid? (sett ett eller flere kryss)

- ☐ Ja, heltid
- ☐ Ja, deltid
- ☐ Arbeidsledig
- ☐ Uførerygdet
- ☐ Hjemmeværende
- ☐ Går på skole, kurs, e.l.
- ☐ Pensjonist
- ☐ Far er død
- ☐ Vet ikke
- ☐ Annet

6) Har du noen gang oppholdt deg 4 uker eller mer sammenhengende i Australia, USA, Argentina eller Sør-Afrika?

○ Ja ○ Nei

Denne informasjonen vises kun i forhåndsvisningen

Følgende betingelser må være oppfylt for at spørsmålet skal vises for respondenten:

Dersom spørsmålet “Har du noen gang oppholdt deg 4 uker eller mer sammenhengende i Australia, USA, Argentina eller Sør-Afrika?” inneholder noen av disse alternativene

- “Ja”

Hvis det har vært flere opphold, oppgi varighet av siste opphold.

7) Hvor lenge varte det siste oppholdet?

- Mindre enn 2 måneder
- 2-6 måneder
- Mer enn 6 måneder

Denne informasjonen vises kun i forhåndsvisningen

Følgende betingelser må være oppfylt for at spørsmålet skal vises for respondenten:

Dersom spørsmålet “Har du noen gang oppholdt deg 4 uker eller mer sammenhengende i Australia, USA, Argentina eller Sør-Afrika?” inneholder noen av disse alternativene

- “Ja”

Hvis det har vært flere opphold, oppgi når du hadde siste opphold.

8) Når var det siste oppholdet?

- Før 1990
- 1991
- 1992
- 1993
- 1994
- 1995
- 1996
- 1997
- 1998
- 1999
- 2000
- 2001
- 2002
- 2003
- 2004
- 2005
- 2006
- 2007
- 2008
- 2009
- 2010
- 2011
- 2012
- 2013

9) Er du i dag?

- Elev i videregående skole
- Lærling/elev i bedrift
- Ikke i videregående opplæring

Denne informasjonen vises kun i forhåndsvisningen

Følgende betingelser må være oppfylt for at spørsmålet skal vises for respondenten:

Dersom spørsmålet “Er du i dag?” inneholder noen av disse alternativene

- “Lærling/elev i bedrift”
- “Elev i videregående skole”

VENNER OG SKOLE

10) Har du vurdert å avbryte eller ta pause fra den videregående opplæringen du er i gang med?

○ Ja ○ Nei

Denne informasjonen vises kun i forhåndsvisningen

Følgende betingelser må være oppfylt for at spørsmålet skal vises for respondenten:

Dersom spørsmålet “Er du i dag?” inneholder noen av disse alternativene

- “Lærling/elev i bedrift”
- “Elev i videregående skole”

11) Hvor sannsynlig er det at du fullfører den utdanningen du er i gang med?

- Liten - kommer til å slutte
- God - kommer sannsynligvis til å fullføre
- Stor - Kommer helt sikkert til å fullføre
- Vet ikke

12) Hvor mange tekstmeldinger (SMS/MMS) sendte du med mobiltelefon i går?

- Ingen
- 1-5 meldinger
- 6-10 meldinger
- 11-20 meldinger
- 21-50 meldinger
- Mer enn 50 meldinger

13) Nedenfor er det noen spørsmål om hvordan du synes du selv er. Kryss av for det som passer best for deg.

	Stemmer svært dårlig	Stemmer nokså dårlig	Stemmer nokså godt	Stemmer svært godt
Jeg synes det er ganske vanskelig å få venner	○	○	○	○
Jeg har mange venner	○	○	○	○
Andre ungdommer har vanskelig for å like meg	○	○	○	○
Jeg er populær blant jevnaldrende	○	○	○	○
Jeg føler at jevnaldrende godtar meg	○	○	○	○

HELSE

14) Hvordan vurderer du din egen helse sånn i alminnelighet?

- Meget god
- God
- Verken god eller dårlig
- Dårlig
- Meget dårlig

15) Hvor ofte har du i løpet av de siste 4 ukene brukt følgende medisiner?

	Ikke brukt siste 4 uker	Sjeldnere enn hver uke	Hver uke, men ikke daglig	Daglig
Smertestillende på resept (f. eks. Paralgin forte, Pinex forte)				
Smertestillende uten resept (f. eks. Paracet, Pinex, Ibux)				
Sovemidler				
Medisin mot depresjon				
Medisiner mot ADHD				
Beroligende medisiner				

16) Har en lege noen gang sagt at du har...

	Ja	Nei	Vet ikke
høysnue eller neseallergi?			
astma?			
barneeksem eller atopisk eksem?			
psoriasis?			

PSYKISKE VANSKER

17) Har du gått i behandling hos psykolog, psykiater eller PP-tjenesten det siste året?

○ Ja ○ Nei

18) Under finner du en liste over ulike problemer. Har du opplevd noe av dette den siste uken (til og med i dag)?

	Ikke plaget	Litt plaget	Ganske mye	Veldig mye
Plutselig frykt uten grunn	○	○	○	○
Føler deg redd eller engstelig	○	○	○	○
Matthet eller svimmelhet	○	○	○	○
Føler deg anspent eller oppjaget	○	○	○	○
Lett for å klandre deg selv	○	○	○	○
Søvnproblemer	○	○	○	○
Nedtrykt, tungsindig	○	○	○	○
Følelse av å være unyttig, lite verdt	○	○	○	○
Følelse av at alt er et slit	○	○	○	○
Følelse av håpløshet med hensyn til framtida	○	○	○	○

19) De følgende spørsmålene handler om hva du følte og gjorde de siste to ukene.

	Ikke riktig	Noen ganger riktig	Riktig
Jeg var lei meg eller ulykkelig	○	○	○
Jeg følte meg så trøtt at jeg bare ble sittende uten å gjøre noen ting	○	○	○
Jeg var veldig rastløs	○	○	○
Jeg var ikke glad for noe	○	○	○
Jeg følte meg lite verdt	○	○	○
Jeg gråt mye	○	○	○
Jeg hatet meg selv	○	○	○
Jeg tenkte at jeg aldri kunne bli så god som andre ungdommer	○	○	○
Jeg følte meg ensom	○	○	○
Jeg tenkte at ingen egentlig var glad i meg	○	○	○
Jeg følte meg som et dårlig menneske	○	○	○
Jeg gjorde alt galt	○	○	○
Jeg syntes det var vanskelig å tenke klart eller å konsentrere meg	○	○	○

De følgende spørmålene handler om hvordan du ser på deg selv.

20) Jeg ser på meg selv som en som...

	Svært uenig 1	2	3	4	5	Svært enig 6
Er pratsom	○	○	○	○	○	○
Har en tendens til å finne feil med andre	○	○	○	○	○	○
Gjør en grundig jobb	○	○	○	○	○	○
Er deprimert, nedstemt	○	○	○	○	○	○
Er orginal, kommer med nye ideer	○	○	○	○	○	○
Er reservert	○	○	○	○	○	○
Er hjelpsom og uegoistisk ovenfor andre	○	○	○	○	○	○
Kan være uforsiktig	○	○	○	○	○	○
Er avslappet, takler stress godt	○	○	○	○	○	○
Er nysgjerrig på mange ting	○	○	○	○	○	○
Er full av energi	○	○	○	○	○	○
Er en kranglefant	○	○	○	○	○	○
Er pålitelig i arbeidet mitt	○	○	○	○	○	○
Kan være anspent	○	○	○	○	○	○
Er skarpsindig, tenker dypt	○	○	○	○	○	○
Skaper mye entusiasme	○	○	○	○	○	○
Er tilgivende av natur	○	○	○	○	○	○
Har en tendens til å være ustrukturert	○	○	○	○	○	○
Bekymrer meg mye	○	○	○	○	○	○
Har livlig fantasi	○	○	○	○	○	○
Har en tendens til å være stillferdig	○	○	○	○	○	○
Er tillitsfull	○	○	○	○	○	○

De følgende spørmålene handler om hvordan du ser på deg selv.

21) Jeg ser på meg selv som en som...

	Svært uenig 1	2	3	4	5	Svært enig 6
Har en tendens til å være lat						
Er følelsesmessig stabil						
Er oppfinnsom						
Er selvhevdende						
Kan være kald og fjern						
Står på til oppgaven er gjennomført						
Kan være humørsyk						
Setter pris på skjønnhet og kunst						
Kan være sjenert og hemmet						
Er hensynsfull og vennlig ovenfor de fleste						
Gjør ting effektivt						
Beholder roen i spente situasjoner						
Foretrekker rutinearbeid						
Er utadvendt og sosial						
Kan noen ganger være uhøflig						
Legger planer og gjennomfører dem						
Blir lett nervøs						
Liker å tenke, leke med ideer						
Har få kunstneriske interesser						
Liker å samarbeide						
Blir lett distrahert						
Har kunnskaper om kunst, musikk, litteratur						

Denne informasjonen vises kun i forhåndsvisningen

Følgende betingelser må være oppfylt for at spørsmålet skal vises for respondenten:

Dersom spørsmålet “Er du:” inneholder noen av disse alternativene

- “Jente”

PUBERTET

Her har vi noen spørsmål om kroppslige forandringer som skjer gjennom ungdomstiden:

22) Har du fått menstruasjon?

- Ja
- Nei

Denne informasjonen vises kun i forhåndsvisningen

Følgende betingelser må være oppfylt for at spørsmålet skal vises for respondenten:

Dersom spørsmålet “Har du fått menstruasjon?” inneholder noen av disse alternativene

- “Ja”

Hvor gammel var du da du fikk menstruasjon første gang?

23) År

- 7 år
- 8 år
- 9 år
- 10 år
- 11 år
- 12 år
- 13 år
- 14 år
- 15 år
- 16 år
- 17 år
- 18 år

Denne informasjonen vises kun i forhåndsvisningen

Følgende betingelser må være oppfylt for at spørsmålet skal vises for respondenten:

Dersom spørsmålet “Har du fått menstruasjon?” inneholder noen av disse alternativene
- “Ja”

24) Måneder

- 1 måned
- 2 måneder
- 3 måneder
- 4 måneder
- 5 måneder
- 6 måneder
- 7 måneder
- 8 måneder
- 9 måneder
- 10 måneder
- 11 måneder

Denne informasjonen vises kun i forhåndsvisningen

Følgende betingelser må være oppfylt for at spørsmålet skal vises for respondenten:

Dersom spørsmålet “Har du fått menstruasjon?” inneholder noen av disse alternativene

- “Ja”

25) Hvis du ser bort fra svangerskap, har du noen gang vært blødningsfri i minst 6 måneder?

- Ja
- Nei

Denne informasjonen vises kun i forhåndsvisningen

Følgende betingelser må være oppfylt for at spørsmålet skal vises for respondenten:

Dersom spørsmålet "Hvis du ser bort fra svangerskap, har du noen gang vært blødningsfri i minst 6 måneder?" inneholder noen av disse alternativene

- "Ja"

26) Hvor mange ganger har du vært blødningsfri i mer enn 6 måneder?

- 1-2
- 3-4
- 5-6
- 7-9
- 10 eller flere

Denne informasjonen vises kun i forhåndsvisningen

Følgende betingelser må være oppfylt for at spørsmålet skal vises for respondenten:

Dersom spørsmålet "Hvis du ser bort fra svangerskap, har du noen gang vært blødningsfri i minst 6 måneder?" inneholder noen av disse alternativene

- "Ja"

27) Hvordan er blødningene dine nå?

- Jeg har regelmessige blødninger
- Jeg har uregelmessige blødninger
- Jeg har ikke hatt blødninger det siste året

Denne informasjonen vises kun i forhåndsvisningen

Følgende betingelser må være oppfylt for at spørsmålet skal vises for respondenten:

Dersom spørsmålet “Er du:” inneholder noen av disse alternativene
- “Gutt”

PUBERTET

28) Når man er tenåring, er det perioder da man vokser raskt. Har du merket at kroppen din har vokst fort (blitt høyere)?

- Nei, den har ikke begynt å vokse
- Ja, den har såvidt begynt å vokse
- Ja, den har helt tydelig begynt å vokse
- Ja, det virker som om jeg er ferdig med å vokse raskt

Denne informasjonen vises kun i forhåndsvisningen

Følgende betingelser må være oppfylt for at spørsmålet skal vises for respondenten:

Dersom spørsmålet “Har du fått menstruasjon?” inneholder noen av disse alternativene

- “Nei”

29) Og hva med hår på kroppen (under armene og i skrittet)? Vil du si at håret på kroppen din har:

- Ikke begynt å vokse enda
- Såvidt begynt å vokse
- Helt tydelig begynt å vokse
- Det virker som om håret på kroppen er utvokst

Denne informasjonen vises kun i forhåndsvisningen

Følgende betingelser må være oppfylt for at spørsmålet skal vises for respondenten:

Dersom spørsmålet “Er du:” inneholder noen av disse alternativene
- “Gutt”

og

Dersom spørsmålet “Og hva med hår på kroppen (under armene og i skrittet)? Vil du si at håret på kroppen din har: ” inneholder noen av disse alternativene
- “Det virker som om håret på kroppen er utvokst”
- “Helt tydelig begynt å vokse”
- “Såvidt begynt å vokse”

30) Hvor gammel var du da du begynte å få hår i skrittet (kjønnshår)?

- 10 år eller yngre
- 11 år
- 12 år
- 13 år
- 14 år
- 15 år
- 16 år
- 17 år
- 18 år
- 19 år
- 20 år

- 21 år
- 22 år
- 23 år
- 24 år
- 25 år
- 26 år
- 27 år
- 28 år
- 29 år
- 30 år eller eldre

Denne informasjonen vises kun i forhåndsvisningen

Følgende betingelser må være oppfylt for at spørsmålet skal vises for respondenten:

Dersom spørsmålet “Er du:” inneholder noen av disse alternativene

- “Gutt”

31) Har du begynt å komme i stemmeskifte?

- Nei, har ikke begynt ennå
- Ja, har såvidt begynt
- Ja, har helt tydelig begynt
- Det virker som om stemmeskifte er ferdig

Denne informasjonen vises kun i forhåndsvisningen

Følgende betingelser må være oppfylt for at spørsmålet skal vises for respondenten:

Dersom spørsmålet “Er du:” inneholder noen av disse alternativene

- “Gutt”

32) Har du begynt å få bart eller skjegg?

- Nei, har ikke begynt ennå
- Ja, har såvidt begynt

- Ja, har helt tydelig begynt
- ja, har fått en god del skjeggvekst

Denne informasjonen vises kun i forhåndsvisningen

Følgende betingelser må være oppfylt for at spørsmålet skal vises for respondenten:

Dersom spørsmålet “Er du:” inneholder noen av disse alternativene

- “Jente”

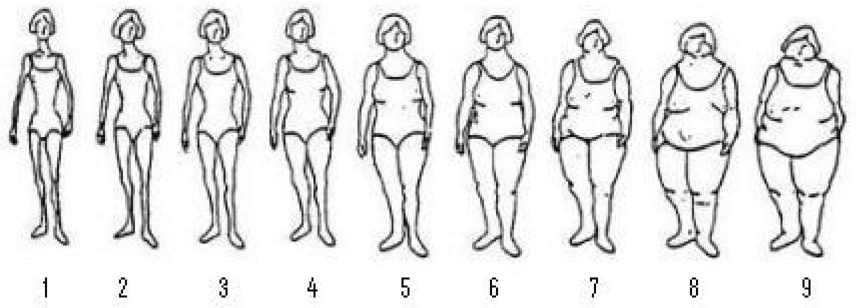

33) Hvilken av disse kroppsfasongene likner mest på din kropp slik du er idag?

- 1
- 2
- 3
- 4
- 5
- 6
- 7
- 8
- 9

Denne informasjonen vises kun i forhåndsvisningen

Følgende betingelser må være oppfylt for at spørsmålet skal vises for respondenten:

Dersom spørsmålet “Er du:” inneholder noen av disse alternativene

- “Gutt”

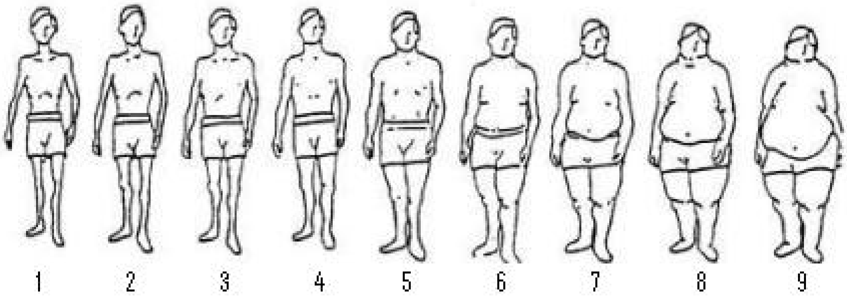

34) Hvilken av disse kroppsfasongene likner mest på din kropp slik du er idag?

- 1
- 2
- 3
- 4
- 5
- 6
- 7
- 8
- 9

35) Gjør du for tiden noe forsøk på å endre kroppsvekten din?

- Nei
- Ja, jeg forsøker å legge på meg
- Ja, jeg forsøker å slanke meg

Denne informasjonen vises kun i forhåndsvisningen

Følgende betingelser må være oppfylt for at spørsmålet skal vises for respondenten:

Dersom spørsmålet "Gjør du for tiden noe forsøk på å endre kroppsvekten din?" inneholder noen av disse alternativene

- "Ja, jeg forsøker å slanke meg"
- "Ja, jeg forsøker å legge på meg"

36) Hvilken vekt vil du være fornøyd med (din trivselsvekt i hele kilo)?

LIVSSTIL

37) Røyker du?

- ○ Nei, aldri
- ○ Før, men ikke nå
- ○ Av og til
- ○ Daglig

Denne informasjonen vises kun i forhåndsvisningen

Følgende betingelser må være oppfylt for at spørsmålet skal vises for respondenten:

Dersom spørsmålet “Røyker du?” inneholder noen av disse alternativene
- “Daglig”
- “Av og til”
- “Før, men ikke nå”

38) Hvor gammel var du da du først begynte å røyke?

- Under 12 år
- 12 år
- 13 år
- 14 år
- 15 år
- 16 år
- 17 år
- 18 år
- 19 år eller mer

Denne informasjonen vises kun i forhåndsvisningen

Følgende betingelser må være oppfylt for at spørsmålet skal vises for respondenten:

Dersom spørsmålet “Røyker du?” inneholder noen av disse alternativene

- “Daglig”
- “Av og til”
- “Før, men ikke nå”

39) Hvor mange sigaretter røyker/røkte du vanligvis i løpet av en uke?

- 1 eller færre
- 2-3
- 4-6
- 7-10
- Mer enn 10

Denne informasjonen vises kun i forhåndsvisningen

Følgende betingelser må være oppfylt for at spørsmålet skal vises for respondenten:

Dersom spørsmålet “Røyker du?” inneholder noen av disse alternativene

- “Daglig”
- “Av og til”
- “Før, men ikke nå”

40) Hvor mange sigaretter røyker/røkte du vanligvis i løpet av en dag?

- 1 eller færre
- 2-3
- 4-6

- 7-10
- Mer enn 10

41) Bruker du snus eller skrå?

- Nei, aldri
- Før, men ikke nå
- Av og til
- Daglig

Denne informasjonen vises kun i forhåndsvisningen

Følgende betingelser må være oppfylt for at spørsmålet skal vises for respondenten:

Dersom spørsmålet “Bruker du snus eller skrå?” inneholder noen av disse alternativene

- “Daglig”
- “Av og til”
- “Før, men ikke nå”

42) Hvor gammel var du da du først begynte å bruke snus eller skrå?

- Under 12 år
- 12 år
- 13 år
- 14 år
- 15 år
- 16 år
- 17 år
- 18 år
- 19 år eller mer

Denne informasjonen vises kun i forhåndsvisningen

Følgende betingelser må være oppfylt for at spørsmålet skal vises for respondenten:

Dersom spørsmålet “Bruker du snus eller skrå?” inneholder noen av disse alternativene

- “Daglig”
- “Av og til”
- “Før, men ikke nå”

43) Hvor mange priser snus/skrå bruker du vanligvis i løpet av en uke?

- 1 eller færre
- 2-3
- 4-6
- 7-10
- Mer enn 10

Denne informasjonen vises kun i forhåndsvisningen

Følgende betingelser må være oppfylt for at spørsmålet skal vises for respondenten:

Dersom spørsmålet “Bruker du snus eller skrå?” inneholder noen av disse alternativene

- “Daglig”
- “Av og til”
- “Før, men ikke nå”

44) Hvor mange priser snus/skrå bruker du per dag?

- 1
- 2-3
- 4-6

- 7-10
- Mer enn 10

45) Hvor ofte drikker du alkohol?

- Aldri
- 1 gang per måned eller sjeldnere
- 2-4 ganger per måned
- 2-3 ganger per uke
- 4 eller flere ganger per uke

Denne informasjonen vises kun i forhåndsvisningen

Følgende betingelser må være oppfylt for at spørsmålet skal vises for respondenten:

Dersom spørsmålet “Hvor ofte drikker du alkohol?” inneholder noen av disse alternativene

- “4 eller flere ganger per uke”
- “2-3 ganger per uke”
- “2-4 ganger per måned”
- “1 gang per måned eller sjeldnere”

46) Hvor mange enheter alkohol (en øl, ett glass vin eller en drink) tar du vanligvis når du drikker?

- 1-2
- 3-4
- 5-6
- 7-9
- 10 eller flere

Denne informasjonen vises kun i forhåndsvisningen

Følgende betingelser må være oppfylt for at spørsmålet skal vises for respondenten:

Dersom spørsmålet “Hvor ofte drikker du alkohol?” inneholder noen av disse alternativene

- “4 eller flere ganger per uke”
- “2-3 ganger per uke”
- “2-4 ganger per måned”

- “1 gang per måned eller sjeldnere”

47) Hvor ofte drikker du 6 eller flere enheter alkohol ved en anledning?

- ○ Aldri
- ○ Sjeldnere enn 1 gang per måned
- ○ 1 gang per måned
- ○ 1 gang per uke
- ○ Daglig eller nesten daglig

FYSISK AKTIVITET

48) Hvilken beskrivelse passer best når det gjelder din fysiske aktivitet på fritiden det siste året?

- Sitter ved PC/TV, leser eller annen stillesittende aktivitet.
- Går, sykler eller beveger deg på annen måte minst 4 timer i uken (her skal du også regne med tur til/fra skolen, shopping, søndagsturer med mer).
- Driver med idrett/trening, tyngre utearbeid, snømåking eller liknende minst 4 timer i uka.
- Trener hardt eller driver konkurranseidrett regelmessig og flere ganger i uka.

49) Hvordan kommer du deg vanligvis til og fra skolen eller arbeid i sommerhalvåret?

- Med bil, motorsykkel/moped
- Med buss
- Med sykkel
- Går
- Ikke i skole eller arbeid

Denne informasjonen vises kun i forhåndsvisningen

Følgende betingelser må være oppfylt for at spørsmålet skal vises for respondenten:

Dersom spørsmålet “Hvordan kommer du deg vanligvis til og fra skolen eller arbeid i sommerhalvåret? ” inneholder noen av disse alternativene

- “Går”
- “Med sykkel”
- “Med buss”
- “Med bil, motorsykkel/moped”

50) Hvor lang tid bruker du vanligvis til og fra skolen eller arbeid (en vei) i sommerhalvåret?

- Mindre enn 5 minutter
- 6 til 15 minutter
- 16 til 30 minutter
- 1/2 til 1 time
- Mer enn 1 time

Denne informasjonen vises kun i forhåndsvisningen

Følgende betingelser må være oppfylt for at spørsmålet skal vises for respondenten:

Dersom spørsmålet “Hvordan kommer du deg vanligvis til og fra skolen eller arbeid i sommerhalvåret? ” inneholder noen av disse alternativene

- “Går”
- “Med sykkel”
- “Med buss”
- “Med bil, motorsykkel/moped”

51) Hvordan kommer du deg vanligvis til og fra skolen eller arbeid i vinterhalvåret?

- Med bil, motorsykkel/moped
- Med buss
- Med sykkel
- Går (til fots eller på ski)

Denne informasjonen vises kun i forhåndsvisningen

Følgende betingelser må være oppfylt for at spørsmålet skal vises for respondenten:

Dersom spørsmålet “Hvordan kommer du deg vanligvis til og fra skolen eller arbeid i sommerhalvåret? ” inneholder noen av disse alternativene

- “Går”
- “Med sykkel”

- “Med buss”
- “Med bil, motorsykkel/moped”

52) Hvor lang tid bruker du vanligvis til og fra skolen eller arbeid (en vei) i vinterhalvåret?

- Mindre enn 5 minutter
- 6 til 15 minutter
- 16 til 30 minutter
- 1/2 til 1 time
- Mer enn 1 time

53) Driver du med idrett eller fysisk aktivitet (f.eks. fotball, dans, løping, sykling, skateboard) utenom skoletid?

- Ja
- Nei

Denne informasjonen vises kun i forhåndsvisningen

Følgende betingelser må være oppfylt for at spørsmålet skal vises for respondenten:

Dersom spørsmålet “Driver du med idrett eller fysisk aktivitet (f.eks. fotball, dans, løping, sykling, skateboard) utenom skoletid?” inneholder noen av disse alternativene

- “Ja”

54) Hvor mange dager i uken driver du med idrett/fysisk aktivitet utenom skoletid?

- Sjeldnere enn 1 dag i uka
- 1 dag i uka
- 2-3 dager i uka
- 4-6 dager i uka
- Omtrent hver dag

Denne informasjonen vises kun i forhåndsvisningen

Følgende betingelser må være oppfylt for at spørsmålet skal vises for respondenten:

Dersom spørsmålet “Driver du med idrett eller fysisk aktivitet (f.eks. fotball, dans, løping, sykling, skateboard) utenom skoletid?” inneholder noen av disse alternativene

- “Ja”

55) Omtrent hvor mange timer per uke bruker du til sammen på idrett/fysisk aktivitet utenom skoletid?

- Omtrent 1/2 time
- Omtrent 1 - 1 1/2 time
- Omtrent 2 - 3 timer
- Omtrent 4 - 6 timer
- 7 timer eller mer

Denne informasjonen vises kun i forhåndsvisningen

Følgende betingelser må være oppfylt for at spørsmålet skal vises for respondenten:

Dersom spørsmålet “Driver du med idrett eller fysisk aktivitet (f.eks. fotball, dans, løping, sykling, skateboard) utenom skoletid?” inneholder noen av disse alternativene

- “Ja”

56) Hvor slitsom er vanligvis idretten/aktiviteten du driver med utenom skoletid?

- Ikke anstrengende
- Litt anstrengende
- Ganske anstrengende
- Meget anstrengende
- Svært anstrengende

Utenom skoletid: Hvor mange timer per dag ser du på PC, TV, DVD og liknende?

57) Hverdager, antall timer per dag:

- Ingen
- Omtrent 1/2 time
- Omtrent 1 - 1 1/2 time
- Omtrent 2 - 3 timer
- Omtrent 4 - 6 timer
- Omtrent 7 - 9 timer
- 10 timer eller mer

58) Fridager (helg, helligdager, ferie), antall timer per dag:

- Ingen
- Omtrent 1/2 time
- Omtrent 1 - 1 1/2 time
- Omtrent 2 - 3 timer
- Omtrent 4 - 6 timer
- Omtrent 7 - 9 timer
- 10 timer eller mer

59) I hvilken grad har andre oppmuntret deg til å være fysisk aktiv

	Svært sjelden/aldri 1	2	3	4	Svært ofte 5
Foreldre/foresatte	○	○	○	○	○
Søsken	○	○	○	○	○
Venner	○	○	○	○	○
Trenere	○	○	○	○	○
Gymlærere	○	○	○	○	○
Nabolaget	○	○	○	○	○

60) Hvordan passer disse utsagnene for deg?

	Helt uenig 1	2	3	4	Helt enig 5
Det er morsommere å drive med trening eller fysisk aktivitet enn å gjøre andre ting...	○	○	○	○	○
Jeg skulle ønske jeg kunne drive mer med trening eller fysisk aktivitet enn det jeg har anledning til å gjøre...	○	○	○	○	○
Jeg føler at jeg er bedre enn de fleste på min alder i idrett/fysisk aktivitet...	○	○	○	○	○
Jeg føler at jeg lett kan holde følge med de andre på min alder når vi driver med idrett/fysisk aktivitet...	○	○	○	○	○

61) Hvordan passer disse utsagnene for deg?

	Helt uenig 1	2	3	4	Helt enig 5
Jeg liker ikke å trene mens noen står å ser på...					
Tilgang til egen garderobe hadde gjort det lettere å trene...					
Jeg blir ubehagelig andpusten, svett eller får vondt i kroppen ved trening...					
Gymtimene er organisert slik at jeg ikke henger med...					
Jeg har ingen å trene sammen med...					
Jeg mangler utstyr for å drive med den aktiviteten jeg har lyst til...					
Jeg har for mange andre oppgaver som gjør at jeg ikke får tid til å trene (f.eks lekser, hjemmeoppgaver)...					
Det mangler egnede haller eller gode uteområder for å drive fysisk aktivitet der jeg bor...					

MATVANER OG KOSTHOLD

62) Hvor ofte pleier du å spise følgende i løpet av en uke?

	Hver dag	4-6 dager i uka	1-3 dager i uka	Sjelden eller aldri
Frokost	○	○	○	○
Middag	○	○	○	○

63) Hvor ofte spiser du matpakke hjemmefra på skole eller arbeid?

- ○ Hver dag
- ○ 3-4 ganger per uke
- ○ 1-2 ganger per uke
- ○ Sjelden eller aldri

64) Hvor ofte spiser du vanligvis disse matvarene?

	Sjelden/ aldri	1-3 ganger per måned	1-3 ganger per uke	4-6 ganger per uke	Hver dag
Ost (alle typer)	○	○	○	○	○
Fet fisk (f.eks. laks, ørret, makrell, sild)	○	○	○	○	○
Mager fisk (f.eks. torsk, sei, hyse)	○	○	○	○	○
Pizza, hamburger eller pølser	○	○	○	○	○
Hermetisert mat (fra metallbokser)	○	○	○	○	○
Godteri (f.eks. sjokolade, drops)	○	○	○	○	○
Snacks og søtsaker (f.eks. potetgull, kake, kjeks, bolle)	○	○	○	○	○
Sukkerfri tyggegummi	○	○	○	○	○

65) Hvor ofte spiser du vanligvis

	Sjelden/ aldri	1-3 ganger per mnd	1-3 ganger per uke	4-6 ganger per uke	1-2 ganger per dag	3-4 ganger per dag	5 eller flere ganger per dag
Frukt	○	○	○	○	○	○	○
Grønnsaker	○	○	○	○	○	○	○

66) Hvor mye drikker du vanligvis av følgende?

	Sjelden/ aldri	1-6 glass per uke	1 glass per dag	2-3 glass per dag	4 glass eller mer per dag
Helmelk, kefir, yoghurt					
Lettmelk, cultura, lettyoghurt					
Skummet melk (sur/søt)					
Ekstra lett melk					
Juice					
Saft med sukker					
Lettsaft, kunstig søtet					
Brus med sukker (1/2 liters flaske = 2 glass)					
Lettbrus, kunstig søtet (1/2 liters flaske = 2 glass)					
Vann					

67) Bruker du følgende kosttilskudd?

	Ja, daglig	Av og til	Nei
Tran, trankapsler, fiskeoljekapsler			
Vitamin- og/eller mineraltilskudd			

SØVN OG SØVNVANER

68) Når pleier du å legge deg for å sove på ukedagene?

- 18.00 eller tidligere
- 18.30
- 19.00
- 19.30
- 20.00
- 20.30
- 21.00
- 21.30
- 22.00
- 22.30
- 23.00
- 23.30
- 00.00
- 00.30
- 01.00
- 01.30
- 02.00 eller senere

69) Når pleier du å legge deg for å sove i helgen?

- 19.00 eller tidligere
- 19.30
- 20.00

- 20.30
- 21.00
- 21.30
- 22.00
- 22.30
- 23.00
- 23.30
- 00.00
- 00.30
- 01.00
- 01.30
- 02.00
- 02.30
- 03.00
- 03.30
- 04.00 eller senere

70) Hvor lenge pleier du å ligge våken før du får sove på ukedagene?

- 1/2 time eller mindre
- 1 time
- 1 1/2 timer
- 2 timer
- 2 1/2 timer
- 3 timer eller mer

71) Hvor lenge pleier du å ligge våken før du får sove i helgen?

- 1/2 time eller mindre
- 1 time
- 1 1/2 timer
- 2 timer
- 2 1/2 timer
- 3 timer eller mer

72) Når pleier du å våkne på ukedagene (endelig oppvåkning)?

- 05.00 eller tidligere
- 05.30
- 06.00
- 06.30
- 07.00
- 07.30
- 08.00
- 08.30
- 09.00
- 09.30
- 10.00 eller senere

73) Når pleier du å våkne i helgen (endelig oppvåkning)?

- 05.00 eller tidligere
- 05.30
- 06.00

- 06.30
- 07.00
- 07.30
- 08.00
- 08.30
- 09.00
- 09.30
- 10.00
- 10.30
- 11.00
- 11.30
- 12.00
- 12.30
- 13.00
- 13.30
- 14.00 eller senere

74) Hvor mange timer sover du vanligvis pr. natt?

- 4 timer eller mindre
- 4 1/2 timer
- 5 timer
- 5 1/2 timer
- 6 timer
- 6 1/2 timer
- 7 timer
- 7 1/2 timer
- 8 timer
- 8 1/2 timer
- 9 timer
- 9 1/2 timer
- 10 timer
- 10 1/2 timer
- 11 timer
- 11 1/2 timer
- 12 timer eller mer

75) Hvor mange timer søvn trenger du pr. natt for å føle deg uthvilt?

- 4 timer eller mindre
- 4 1/2 timer
- 5 timer
- 5 1/2 timer

- 6 timer
- 6 1/2 timer
- 7 timer
- 7 1/2 timer
- 8 timer
- 8 1/2 timer
- 9 timer
- 9 1/2 timer
- 10 timer
- 10 1/2 timer
- 11 timer
- 11 1/2 timer
- 12 timer eller mer

76) Synes du at du får tilstrekkelig med søvn?

- Ja, absolutt tilstrekkelig
- Ja, stort sett tilstrekkelig
- Nei, noe utilstrekkelig
- Nei, klart utilstrekkelig
- Nei, langt fra tilstrekkelig

77) I løpet av den siste måneden, hvor mange dager pr. uke har du

	0 dager	1 dag	2 dager	3 dager	4 dager	5 dager	6 dager	7 dager
brukt mer enn 30 minutter for å sovne etter at lysene ble slukket?								
vært våken mer enn 30 minutter innimellom søvnen?								
våknet mer enn 30 mintter tidligere enn du ønsket å gjøre uten å få sove igjen?								
følt deg for lite uthvilt etter å ha sovet?								
vært så søvnig/trett at det har gått ut over skole/jobb eller privatlivet?								
vært misfornøyd med søvnen din?								
hatt vansker med å sovne før kl 02:00?								
hatt vansker med å våkne om morgenen?								
har du forsovet deg til skolen, arbeid eller avtaler?								

Følsomhet for støy

78) Hvor enig eller uenig er du i utsagnene? Sett kryss for det svaralternativet som passer best for hvert utsagn

	Helt uenig	Ganske uenig	Litt uenig	Litt enig	Ganske enig	Helt enig
Jeg vekkes lett av støy	○	○	○	○	○	○
Jeg venner meg til de fleste lyder uten store problemer	○	○	○	○	○	○
Det er vanskelig for meg å slappe av på et sted med mye støy	○	○	○	○	○	○
Jeg er flink til å konsentrere meg uansett hva som skjer rundt meg	○	○	○	○	○	○
Jeg blir sint på folk som lager støy som hindrer meg i å sovne eller å få gjort jobben min	○	○	○	○	○	○
Jeg er følsom for støy	○	○	○	○	○	○

SOLING

79) Hva skjer med huden din hvis du soler deg om sommeren?

- Alltid rød, aldri brun
- Nesten alltid rød, av og til brun
- Nesten alltid brun, av og til rød
- Alltid brun, aldri rød

80) Har du vært i Syden-/solingsferie de siste 2 måneder?

- Ja
- Nei

81) Har du tatt solarium i løpet av de siste 4 ukene?

- Nei
- Ja, en gang
- Ja, flere ganger

SMERTER

82) Har du langvarige eller stadig tilbakevendende smerter som har vart i 3 måneder eller mer?

○ Ja ○ Nei

Denne informasjonen vises kun i forhåndsvisningen

Følgende betingelser må være oppfylt for at spørsmålet skal vises for respondenten:

Dersom spørsmålet “Har du langvarige eller stadig tilbakevendende smerter som har vart i 3 måneder eller mer?” inneholder noen av disse alternativene

- “Ja”

83) Hvor ofte har du vanligvis disse smertene?

- Hele tiden, uten opphør
- Hver dag, men ikke hele tiden
- Hver uke, men ikke hver dag
- Sjeldnere enn hver uke

Denne informasjonen vises kun i forhåndsvisningen

Følgende betingelser må være oppfylt for at spørsmålet skal vises for respondenten:

Dersom spørsmålet “Har du langvarige eller stadig tilbakevendende smerter som har vart i 3 måneder eller mer?” inneholder noen av disse alternativene

- “Ja”

84) Hvor lenge har du hatt disse smertene? (Dersom du har flere typer smerte, svar for den som har vart lengst)

- 3 - 6 måneder
- 6 - 12 måneder
- 1-2 år
- 3-6 år
- Mer enn 6 år

Denne informasjonen vises kun i forhåndsvisningen

Følgende betingelser må være oppfylt for at spørsmålet skal vises for respondenten:

Dersom spørsmålet “Har du langvarige eller stadig tilbakevendende smerter som har vart i 3 måneder eller mer?” inneholder noen av disse alternativene

- “Ja”

Hvor er det vondt?

(Kryss av på alle aktuelle steder)

	Venstre side	Høyre side
Skulder	☐	☐
Arm/albue	☐	☐
Hånd	☐	☐
Hofte	☐	☐
Lår/kne/legg	☐	☐
Ankel/fot	☐	☐

Denne informasjonen vises kun i forhåndsvisningen

Følgende betingelser må være oppfylt for at spørsmålet skal vises for respondenten:

Dersom spørsmålet “Har du langvarige eller stadig tilbakevendende smerter som har vart i 3 måneder eller mer?” inneholder noen av disse alternativene

- “Ja”

- Hode/ansikt ☐
- Kjeve/kjeveledd ☐
- Nakke ☐
- Øvre del av ryggen ☐
- Korsryggen ☐
- Bryst ☐
- Mage ☐
- Underliv/kjønnsorganer ☐

Denne informasjonen vises kun i forhåndsvisningen

Følgende betingelser må være oppfylt for at spørsmålet skal vises for respondenten:

Dersom spørsmålet “Har du langvarige eller stadig tilbakevendende smerter som har vart i 3 måneder eller mer?” inneholder noen av disse alternativene

- “Ja”

87) Hva mener du er årsaken til smertene? (flere svar mulig)

- ☐ PC-bruk, dataspill og lignende
- ☐ Idrettsskade
- ☐ Ulykke/skade
- ☐ Kirurgisk inngrep/operasjon
- ☐ Migrene/hodepine
- ☐ Medfødt sykdom
- ☐ Tannproblemer
- ☐ Whiplash
- ☐ Prolaps (skiveutglidning i ryggen)
- ☐ Annet ryggproblem
- ☐ Nerveskade
- ☐ Mage- eller tarmsykdom

Denne informasjonen vises kun i forhåndsvisningen

Følgende betingelser må være oppfylt for at spørsmålet skal vises for respondenten:

Dersom spørsmålet "Har du langvarige eller stadig tilbakevendende smerter som har vart i 3 måneder eller mer?" inneholder noen av disse alternativene

- "Ja"

Hvis du har langvarige smerter flere steder i kroppen, gjelder de 4 neste spørsmålene smerten som plager deg mest.

Dersom du har flere typer smerte, svar den som plager deg mest.

88) Hvor sterke vil du si at smertene vanligvis er?

- 0 Ingen smerte
- 1
- 2
- 3
- 4
- 5
- 6
- 7
- 8
- 9
- 10 Verst tenkelige smerte

Denne informasjonen vises kun i forhåndsvisningen

Følgende betingelser må være oppfylt for at spørsmålet skal vises for respondenten:

Dersom spørsmålet “Har du langvarige eller stadig tilbakevendende smerter som har vart i 3 måneder eller mer?” inneholder noen av disse alternativene

- “Ja”

89) Hvor sterke er smertene når de er på sitt sterkeste?

- 0 Ingen smerte
- 1
- 2
- 3
- 4
- 5
- 6
- 7
- 8
- 9
- 10 Verst tenkelige smerte

Denne informasjonen vises kun i forhåndsvisningen

Følgende betingelser må være oppfylt for at spørsmålet skal vises for respondenten:

Dersom spørsmålet “Har du langvarige eller stadig tilbakevendende smerter som har vart i 3 måneder eller mer?” inneholder noen av disse alternativene

- “Ja”

90) I hvor stor grad påvirker smertene søvnen din?

- 0 Ingen påvirkning
- 1
- 2
- 3
- 4
- 5
- 6
- 7
- 8
- 9
- 10 Umulig å få sove på grunn av smertene

Denne informasjonen vises kun i forhåndsvisningen

Følgende betingelser må være oppfylt for at spørsmålet skal vises for respondenten:

Dersom spørsmålet “Har du langvarige eller stadig tilbakevendende smerter som har vart i 3 måneder eller mer?” inneholder noen av disse alternativene

- “Ja”

91) I hvor stor grad hindrer smertene deg i å utføre vanlige aktiviteter hjemme og på skolen?

- 0 Påvirker ikke vanlige aktiviteter
- 1
- 2
- 3
- 4
- 5
- 6
- 7
- 8
- 9
- 10 Kan ikke gjøre noe på grunn av smertene

92) Får du smerter i muskler og ledd når du har feber?

Ja

Nei

➯

Denne informasjonen vises kun i forhåndsvisningen

Følgende betingelser må være oppfylt for at spørsmålet skal vises for respondenten:

Dersom spørsmålet “Får du smerter i muskler og ledd når du har feber?” inneholder noen av disse alternativene

- “Ja”

93) Hvor sterke er febersmertene vanligvis?

- 0 Ingen smerte
- 1
- 2
- 3
- 4
- 5
- 6
- 7
- 8
- 9
- 10 Verst tenkelige smerte

MAGE- OG TARMPROBLEMER

94) I løpet av de siste 2 månedene: Hvor ofte har du hatt smerte eller ubehag i magen?

- Aldri
- 1-3 ganger i måneden
- En gang i uka
- Flere ganger i uka
- Hver dag

Denne informasjonen vises kun i forhåndsvisningen

Følgende betingelser må være oppfylt for at spørsmålet skal vises for respondenten:

Dersom spørsmålet “I løpet av de siste 2 månedene: Hvor ofte har du hatt smerte eller ubehag i magen?” inneholder noen av disse alternativene

- “Hver dag”
- “Flere ganger i uka”
- “En gang i uka”
- “1-3 ganger i måneden”

95) Hvor lenge har du vært plaget av smerte eller ubehag i magen?

- Mindre enn 1 måned
- 2 måneder
- 3 måneder
- 4-11 måneder
- Ett år eller mer

Denne informasjonen vises kun i forhåndsvisningen

Følgende betingelser må være oppfylt for at spørsmålet skal vises for respondenten:

Dersom spørsmålet “Hvor lenge har du vært plaget av smerte eller ubehag i magen?” inneholder noen av disse alternativene

- “Ett år eller mer”
- “4-11 måneder”
- “3 måneder”
- “2 måneder”

96) I hvilken del av magen er det du har hatt smerte eller ubehag? (kryss av for alt som passer)

- ☐ Over navlen
- ☐ Rundt navlen
- ☐ Nedenfor navlen

Denne informasjonen vises kun i forhåndsvisningen

Følgende betingelser må være oppfylt for at spørsmålet skal vises for respondenten:

Dersom spørsmålet “Hvor lenge har du vært plaget av smerte eller ubehag i magen?” inneholder noen av disse alternativene

- “Ett år eller mer”
- “4-11 måneder”
- “3 måneder”
- “2 måneder”

97) Når du har smerter eller ubehag i magen, hvor lenge varer det vanligvis?

- Mindre enn 1 time
- 1-2 timer
- 3-4 timer
- Mesteparten av dagen
- Hele døgnet

Denne informasjonen vises kun i forhåndsvisningen

Følgende betingelser må være oppfylt for at spørsmålet skal vises for respondenten:

Dersom spørsmålet “Hvor lenge har du vært plaget av smerte eller ubehag i magen?” inneholder noen av disse alternativene

- “Ett år eller mer”
- “4-11 måneder”
- “3 måneder”
- “2 måneder”

98) Når du har smerte eller ubehag i magen, hvor sterke smerter har du vanligvis?

- 0 Ingen smerte
- 1
- 2
- 3
- 4
- 5
- 6

- 7
- 8
- 9
- 10 Verst tenkelige smerte

Denne informasjonen vises kun i forhåndsvisningen

Følgende betingelser må være oppfylt for at spørsmålet skal vises for respondenten:

Dersom spørsmålet “Hvor lenge har du vært plaget av smerte eller ubehag i magen?” inneholder noen av disse alternativene

- “Ett år eller mer”
- “4-11 måneder”
- “3 måneder”
- “2 måneder”

99) Når du har smerter eller ubehag i magen, hvor ofte blir det bedre etter at du har hatt avføring?

- Sjelden eller aldri
- En del ganger
- For det meste/hver gang

Denne informasjonen vises kun i forhåndsvisningen

Følgende betingelser må være oppfylt for at spørsmålet skal vises for respondenten:

Dersom spørsmålet “Hvor lenge har du vært plaget av smerte eller ubehag i magen?” inneholder noen av disse alternativene

- “Ett år eller mer”
- “4-11 måneder”
- “3 måneder”
- “2 måneder”

100) Når du har smerter eller ubehag i magen, hvor ofte skjer det i forbindelse med at du..

	Sjelden eller aldri	En del ganger	For det meste
har fastere eller mer klumpete avføring enn vanlig?	○	○	○
har løsere eller mer vannaktig avføring enn vanlig?	○	○	○
hadde avføring oftere enn vanlig?	○	○	○
hadde avføring sjeldnere enn vanlig?	○	○	○

HODEPINE

101) Har du vært plaget av hodepine det siste året?

- Ja
- Nei

Denne informasjonen vises kun i forhåndsvisningen

Følgende betingelser må være oppfylt for at spørsmålet skal vises for respondenten:

Dersom spørsmålet “Har du vært plaget av hodepine det siste året?” inneholder noen av disse alternativene

- “Ja”

102) Hva slags hodepine er du plaget av? (Du kan sette flere kryss)

☐ Migrene ☐ Annen hodepine ☐ Vet ikke

Denne informasjonen vises kun i forhåndsvisningen

Følgende betingelser må være oppfylt for at spørsmålet skal vises for respondenten:

Dersom spørsmålet “Har du vært plaget av hodepine det siste året?” inneholder noen av disse alternativene

- “Ja”

103) Omtrent hvor mange dager per måned har du hodepine?

○ Mindre enn 1 dag

○ 1-6 dager

○ 7-14 dager

○ Mer enn 14 dager

Denne informasjonen vises kun i forhåndsvisningen

Følgende betingelser må være oppfylt for at spørsmålet skal vises for respondenten:

Dersom spørsmålet “Har du vært plaget av hodepine det siste året?” inneholder noen av disse alternativene

- “Ja”

104) Er hodepinen vanligvis:

	Ja	Nei
Bankende/dunkende smerte	○	○
Pressende smerte	○	○
Ensidig smerte (høyre eller venstre)	○	○

Denne informasjonen vises kun i forhåndsvisningen

Følgende betingelser må være oppfylt for at spørsmålet skal vises for respondenten:

Dersom spørsmålet “Har du vært plaget av hodepine det siste året?” inneholder noen av disse alternativene

- “Ja”

105) Hvor lenge varer hodepinen vanligvis?

- Mindre enn 4 timer
- 4 timer - 1 døgn
- 1-3 døgn
- Mer enn 3 døgn

Denne informasjonen vises kun i forhåndsvisningen

Følgende betingelser må være oppfylt for at spørsmålet skal vises for respondenten:

Dersom spørsmålet “Har du vært plaget av hodepine det siste året?” inneholder noen av disse alternativene

- “Ja”

106) Før eller under hodepinen, kan du da ha forbigående:

	Ja	Nei
Synsforstyrrelse? (takkede linjer, flimring, tåkesyn, lysglimt)		

	Ja	Nei
Nummenhet i halve ansiktet eller i hånden?	○	○
Forverring ved moderat fysisk aktivitet?	○	○
Kvalme og/eller oppkast?	○	○

Nedenfor er det fire spørsmål om hvordan du opplever det er å gå til tannlege. Les hvert spørsmål og velg det svaralternativet som du synes passer best for deg.

107) Dersom du skulle gå til tannlegen i morgen, hva ville du føle?

- Jeg ville se frem til det som en ganske hyggelig opplevelse
- Det ville være det samme for meg, ikke bety noe
- Det ville gjøre meg litt urolig
- Jeg ville bli redd for at det skulle bli ubehagelig og vondt
- Jeg ville bli svært redd med tanke på hva tannlegen kanskje skulle gjøre

108) Når du venter på tannlegens venteværelse, hvordan føler du deg da?

- Avslappet
- Litt urolig
- Anspent, nervøs
- Redd, engstelig
- Så redd at jeg av og til begynner å svette eller nesten føler meg syk

109) Når du sitter i tannlegestolen og venter på at tannlegen skal begynne behandlingen, hvordan føler du deg da?

- Avslappet
- Litt urolig
- Anspent, nervøs
- Redd, engstelig
- Så redd at jeg av og til begynner å svette eller nesten føler meg syk

110) Tenk at du sitter i tannlegestolen og skal få tennene renset og pusset. Mens du sitter og venter på at tannlege skal finne frem instrumentene som brukes til å skrape og pusse med, hvordan føler du deg da?

- Avslappet
- Litt urolig
- Anspent, nervøs
- Redd, engstelig
- Så redd at jeg av og til begynner å svette eller nesten føler meg syk

HØRSEL

111) Har du et hørseltap som du vet om?

- Nei
- Ja
- Kanskje

Denne informasjonen vises kun i forhåndsvisningen

Følgende betingelser må være oppfylt for at spørsmålet skal vises for respondenten:

Dersom spørsmålet “Har du et hørseltap som du vet om?” inneholder noen av disse alternativene
- “Ja”

112) Er hørseltapet bekreftet av lege eller annet helsepersonell?

○ Nei ○ Ja

Denne informasjonen vises kun i forhåndsvisningen

Følgende betingelser må være oppfylt for at spørsmålet skal vises for respondenten:

Dersom spørsmålet “Har du et hørseltap som du vet om?” inneholder noen av disse alternativene
- “Ja”

113) Bruker du høreapparat?

○ Nei ○ Ja

114) Har du øresus?

- Aldri
- Sjelden
- Ofte

Denne informasjonen vises kun i forhåndsvisningen

Følgende betingelser må være oppfylt for at spørsmålet skal vises for respondenten:

Dersom spørsmålet “Har du øresus?” inneholder noen av disse alternativene

- “Ofte”
- “Sjelden”

115) Hvor ofte har du øresus?

- Hele tiden, uten opphør
- Hver dag, men ikke hele tiden
- Hver uke, men ikke hver dag
- Sjeldnere enn hver uke

Denne informasjonen vises kun i forhåndsvisningen

Følgende betingelser må være oppfylt for at spørsmålet skal vises for respondenten:

Dersom spørsmålet “Har du øresus?” inneholder noen av disse alternativene

- “Ofte”
- “Sjelden”

116) Hvor lenge varer vanligvis periodene med øresus?

- Mindre enn 10 minutter
- 10 minutter - 1 time
- Mer enn 1 time

Denne informasjonen vises kun i forhåndsvisningen

Følgende betingelser må være oppfylt for at spørsmålet skal vises for respondenten:

Dersom spørsmålet “Har du øresus?” inneholder noen av disse alternativene

- “Ofte”
- “Sjelden”

117) Når får du vanligvis øresus?

- Etter sterke lyder
- Når det er stille
- Vet aldri når

Denne informasjonen vises kun i forhåndsvisningen

Følgende betingelser må være oppfylt for at spørsmålet skal vises for respondenten:

Dersom spørsmålet “Har du øresus?” inneholder noen av disse alternativene

- “Ofte”
- “Sjelden”

118) Noen bryr seg ikke om lyden, for andre oppleves det svært plagsomt å ha øresus. Angi hvor plaget du er av øresusen.

- 0 Ingen plager
- 1
- 2
- 3
- 4
- 5
- 6
- 7
- 8
- 9
- 10 Verst tenkelige plager

Denne informasjonen vises kun i forhåndsvisningen

Følgende betingelser må være oppfylt for at spørsmålet skal vises for respondenten:

Dersom spørsmålet “Har du øresus?” inneholder noen av disse alternativene

- “Ofte”
- “Sjelden”

119) På hvilket øre har du vanligvis øresus?

- Bare høyre
- Bare venstre
- Begge, men mest høyre
- Begge, men mest venstre
- Like mye på begge

Denne informasjonen vises kun i forhåndsvisningen

Følgende betingelser må være oppfylt for at spørsmålet skal vises for respondenten:

Dersom spørsmålet “Har du øresus?” inneholder noen av disse alternativene

- “Sjelden”

120) Omtrent hvor gammel var du når du begynte å ha øresus?

- 1 år
- 2 år
- 3 år
- 4 år
- 5 år
- 6 år
- 7 år
- 8 år
- 9 år
- 10 år
- 11 år
- 12 år
- 13 år
- 14 år
- 15 år

- 16 år
- 17 år
- 18 år
- 19 år
- 20 år eller mer

Denne informasjonen vises kun i forhåndsvisningen

Følgende betingelser må være oppfylt for at spørsmålet skal vises for respondenten:

Dersom spørsmålet "Har du øresus?" inneholder noen av disse alternativene

- "Ofte"

121) Omtrent hvor gammel var du når du begynte å ha øresus ofte?

- 1 år
- 2 år
- 3 år
- 4 år
- 5 år
- 6 år
- 7 år
- 8 år
- 9 år
- 10 år
- 11 år
- 12 år
- 13 år
- 14 år
- 15 år

- 16 år
- 17 år
- 18 år
- 19 år
- 20 år eller mer

SYKEHUS OG INFEKSJONER

122) Har du vært innlagt som pasient på sykehus i løpet av de siste 12 månedene?

○ Ja ○ Nei

123) Arbeider noen du bor sammen med i helsevesenet (sykehus, sykehjem, hjemmetjenesten, legekontor, helsestasjon)?

○ Ja ○ Nei

124) Har du tidligere fått fjernet mandlene?

- Ja
- Nei
- Vet ikke

Denne informasjonen vises kun i forhåndsvisningen

Følgende betingelser må være oppfylt for at spørsmålet skal vises for respondenten:

Dersom spørsmålet “Har du tidligere fått fjernet mandlene?” inneholder noen av disse alternativene

- “Ja”

125) Jeg fikk fjernet mandlene fordi jeg hadde

- halsbetennelse som kom og gikk
- halsbetennelse og vondt i halsen og/eller dårlig ånde hele tiden
- store mandler og trang hals (dette kan gi svelgproblemer, snorking, pustestopp)
- både halsbetennelse og store mandler
- vet ikke

Denne informasjonen vises kun i forhåndsvisningen

Følgende betingelser må være oppfylt for at spørsmålet skal vises for respondenten:

Dersom spørsmålet “Har du tidligere fått fjernet mandlene?” inneholder noen av disse alternativene

- “Nei”

126) Jeg har nå

- ingen plager fra halsen
- plaget med halsbetennelse som kommer og går
- konstante plager med halsbetennelse og vondt i halsen og/eller dårlig ånde
- store mandler og trang hals (dette kan gi svelgproblemer, snorking, pustestopp)
- plaget med både halsbetennelse og store mandler

TANNHELSE

127) Hvor ofte pusser du vanligvis tennene dine?

- Sjeldnere enn 1 gang per uke
- 1 gang per uke
- 2-3 ganger per uke
- 4-6 ganger per uke
- 1 gang daglig
- 2 eller flere ganger daglig

128) Hvor ofte bruker du noen av følgende hjelpemidler?

	Daglig	Noen ganger i uka	Noen ganger i måneden	Sjelden/aldri
Fluor tannkrem				
Tanntråd				
Tannstikker				
Fluortabletter				
Fluor skyllevæske				

129) Hvor ofte kontrollerte foreldrene dine eller dine foresatte at du hadde pusset tennene dine da du var yngre?

- Ofte
- Omtrent daglig
- Av og til
- Sjelden/aldri

130) Hvordan vurderer du din egen tannhelse?

- Meget god
- God
- Verken god eller dårlig
- Dårlig
- Meget dårlig

131) Hvorfor er fluor tilsatt i tannkrem?

- Behagelig smak
- Gir god ånde
- Hindrer hull i tennene
- Gir hvite tenner

132) Har du følt at tannlegen/tannpleieren ikke tar seg tid til å forklare eller svare på spørsmål?

- Ja, ofte
- Ja, av og til
- Nei

133) Er du fornøyd med tannstillingen din i fronten?

- Veldig fornøyd
- Fornøyd
- Ganske fornøyd
- Verken fornøyd eller misfornøyd
- Ganske misfornøyd
- Misfornøyd
- Veldig misfornøyd

134) Prøver du å unngå å smile på grunn av dine tenners utseende?

- Aldri
- Veldig sjelden
- Sjelden
- Vanskelig å si
- Av og til
- Ganske ofte
- Ofte

135) Ønsker du tannregulering for å få rettet opp tennene dine?

- Ja, absolutt
- Ja
- Ja, kanskje
- Verken ja eller nei

- Tror ikke det
- Nei
- Absolutt ikke

136) Har du hatt fast tannregulering/streng?

○ Ja ○ Nei

137) Har du hatt avtagbar plate?

○ Ja ○ Nei

138) Har du hatt tannregulering siden forrige gang du deltok i Fit Futures undersøkelsen?

- Nei
- Ja
- Har ikke deltatt tidligere

Denne informasjonen vises kun i forhåndsvisningen

Følgende betingelser må være oppfylt for at spørsmålet skal vises for respondenten:

Dersom spørsmålet “Har du hatt fast tannregulering/streng?” inneholder noen av disse alternativene

- “Ja”

139) Hadde du allergiske reaksjoner i forbindelse med tannreguleringen?

○ Ja ○ Nei

140) Hvor smertefullt, jevnt over, synes du det er å gå til tannlegen?

- 0 Ingen smerte
- 1
- 2
- 3
- 4
- 5
- 6
- 7
- 8
- 9
- 10 Verst tenkelige smerte

141) Har du latt være å møte opp til en tannlegetime pga frykt for tannbehandling?

- Ja
- Nei

Ta stilling til følgende påstander:

142) Tannpuss er svært viktig for meg når jeg skal

	Helt uenig	Uenig	Enig	Helt enig
ut med venner på ungdomsklubb, diskotek osv.				
møte en kjæreste				
på skolen				
møte min beste venn/venninne				
delta i sport eller drive med hobbyer				
til tannlegen				

143) Tannpuss er svært viktig for at jeg skal

	Helt uenig	Uenig	Enig	Helt enig
føle meg frisk				
unngå hull i tennene				
unngå at tennene får en stygg farge				
få frisk pust				
beholde sunt tannkjøtt				
få bedre utseende				

144) jeg synes det ville være pinlig dersom det ble hull i

	Helt uenig	Uenig	Enig	Helt enig
mine egne tenner				
min mors tenner				
min fars tenner				
min venn/venninnes tenner				

145) Tannpuss er svært viktig for at jeg skal få

	Helt uenig	Uenig	Enig	Helt enig
mine foreldres anerkjennelse				
mine venners anerkjennelse				

Ta stilling til følgende utsagn

146) Hvor sikker er du på at du vil pusse tennene 2 ganger om dagen i 2 minutter med fluortannkrem i følgende situasjoner

	Svært sikker	Ganske sikker	Noe usikker	Ganske usikker
Når du er trøtt om kvelden	○	○	○	○
Når du har mye å gjøre (mye lekser, eksamener)	○	○	○	○
Når du har skoleferie	○	○	○	○
Når du er trøtt på morgenen	○	○	○	○
Når du føler deg syk (hodepine)	○	○	○	○

147) Hvor sikker er du på at du er villig til avstå fra sukkerholdige drikker som brus, juice og saft til andre tider enn ved lunsj eller middag?

- ○ Svært sikker
- ○ Ganske sikker
- ○ Noe usikker
- ○ Svært usikker

148) Jeg har til hensikt å pusse tennene 2 ganger om dagen i minst 2 minutter med fluortannkrem hver dag

- ○ Helt enig
- ○ Enig
- ○ Uenig
- ○ Helt uenig

149) Hvor fornøyd er du med din tannhelse?

- Svært misfornøyd
- Misfornøyd
- Verken eller
- Fornøyd
- Svært fornøyd

150) Har du vært, eller er du, plaget med sur smak i munnen eller sure oppstøt?

○ Nei ○ Ja

Denne informasjonen vises kun i forhåndsvisningen

Følgende betingelser må være oppfylt for at spørsmålet skal vises for respondenten:

Dersom spørsmålet “Har du vært, eller er du, plaget med sur smak i munnen eller sure oppstøt?” inneholder noen av disse alternativene

- “Ja”

151) Hvor ofte har du vært, eller er du, plaget med sur smak i munnen eller sure oppstøt?

- Daglig
- Noen ganger i uken
- Månedlig
- Sjelden eller aldri

Denne informasjonen vises kun i forhåndsvisningen

Følgende betingelser må være oppfylt for at spørsmålet skal vises for respondenten:

Dersom spørsmålet “Har du vært, eller er du, plaget med sur smak i munnen eller sure oppstøt?” inneholder noen av disse alternativene

- “Ja”

152) Hvor lenge har det vart?

- Uker

- Måneder
- Flere år

153) Har du vært, eller er du, plaget med oppkast?

○ Nei ○ Ja

Denne informasjonen vises kun i forhåndsvisningen

Følgende betingelser må være oppfylt for at spørsmålet skal vises for respondenten:

Dersom spørsmålet “Har du vært, eller er du, plaget med oppkast?” inneholder noen av disse alternativene

- “Ja”

154) Hvor ofte har du vært, eller er du, plaget med oppkast?

- Daglig
- Noen ganger i uken
- Månedlig
- Sjelden eller aldri

Denne informasjonen vises kun i forhåndsvisningen

Følgende betingelser må være oppfylt for at spørsmålet skal vises for respondenten:

Dersom spørsmålet “Har du vært, eller er du, plaget med oppkast?” inneholder noen av disse alternativene

- “Ja”

155) Hvor lenge har det vart?

- Uker
- Måneder

- Flere år

ASTMA OG PUSTEBESVÆR

156) Har du - de siste 12 månedene - hatt pipende eller hvesende pust?

○ Nei ○ Ja

Denne informasjonen vises kun i forhåndsvisningen

Følgende betingelser må være oppfylt for at spørsmålet skal vises for respondenten:

Dersom spørsmålet “Har du - de siste 12 månedene - hatt pipende eller hvesende pust?” inneholder noen av disse alternativene

- “Ja”

157) Hvor mange ganger har du hatt disse plagene de siste 12 månedene ?

- 1-3 ganger
- 4-12 ganger
- Mer enn 12 ganger

Denne informasjonen vises kun i forhåndsvisningen

Følgende betingelser må være oppfylt for at spørsmålet skal vises for respondenten:

Dersom spørsmålet “Har du - de siste 12 månedene - hatt pipende eller hvesende pust?” inneholder noen av disse alternativene

- “Ja”

158) Har du - de siste 12 månedene - unnlatt å gjøre ting du vil gjøre pga pipende eller hvesende pust?

- Nei

- ○ Ja

➯

Denne informasjonen vises kun i forhåndsvisningen

Følgende betingelser må være oppfylt for at spørsmålet skal vises for respondenten:

Dersom spørsmålet “Har du - de siste 12 månedene - unnlatt å gjøre ting du vil gjøre pga pipende eller hvesende pust?” inneholder noen av disse alternativene

- “Ja”

159) Hvor mye har pipende eller hvesende pust hindret deg fra å gjøre ting du har villet gjøre de siste 12 månedene?

- Lite
- Moderat
- Ganske mye
- Mye

Denne informasjonen vises kun i forhåndsvisningen

Følgende betingelser må være oppfylt for at spørsmålet skal vises for respondenten:

Dersom spørsmålet “Har du - de siste 12 månedene - hatt pipende eller hvesende pust?” inneholder noen av disse alternativene

- “Ja”

160) Har du - de siste 12 månedene - hatt vanskelig for å sove, eller våknet pga pipende eller hvesende pust?

- Nei
- Mindre enn en gang i uken
- 1 eller flere ganger i uken

Denne informasjonen vises kun i forhåndsvisningen

Følgende betingelser må være oppfylt for at spørsmålet skal vises for respondenten:

Dersom spørsmålet “Har du - de siste 12 månedene - hatt pipende eller hvesende pust?” inneholder noen av disse alternativene

- “Ja”

161) Har du - de siste 12 månedene - vært borte fra skolen pga pipende eller hvesende pust?

- Nei
- Ja

Denne informasjonen vises kun i forhåndsvisningen

Følgende betingelser må være oppfylt for at spørsmålet skal vises for respondenten:

Dersom spørsmålet “Har du - de siste 12 månedene - vært borte fra skolen pga pipende eller hvesende pust?” inneholder noen av disse alternativene

- “Ja”

162) Hvor mange dager har du vært borte fra skolen pga pipende eller hvesende pust de siste 12 månedene?

- Mindre enn 5 dager
- 5-10 dager
- Mer enn 10 dager

Denne informasjonen vises kun i forhåndsvisningen

Følgende betingelser må være oppfylt for at spørsmålet skal vises for respondenten:

Dersom spørsmålet “Har du - de siste 12 månedene - hatt pipende eller hvesende pust?” inneholder noen av disse alternativene

- “Ja”

163) Har du - de siste 12 månedene - hatt så store plager med pipende eller hvesende pust, at du har hatt behov for å ta nye åndedrag midt i en setning?

○ Nei ○ Ja

164) Har du - de siste 12 månedene - hatt pustebesvær (hatt tungt for å puste, kjent deg tett i brystet, hatt pipende eller hvesende pust)?

- Nei
- Ja

Denne informasjonen vises kun i forhåndsvisningen

Følgende betingelser må være oppfylt for at spørsmålet skal vises for respondenten:

Dersom spørsmålet “Har du - de siste 12 månedene - hatt pustebesvær (hatt tungt for å puste, kjent deg tett i brystet, hatt pipende eller hvesende pust)?” inneholder noen av disse alternativene

- “Ja”

165) Dersom du har hatt pustebesvær eller pipende eller hvesende pust de siste 12 månedene, hvor tungt opplevde du at det var å puste? (Marker med et kryss på linjen)

- 0 Ikke tungt i det hele tatt
- 1
- 2
- 3
- 4
- 5
- 6
- 7
- 8
- 9
- 10 Verst tenkbar

Denne informasjonen vises kun i forhåndsvisningen

Følgende betingelser må være oppfylt for at spørsmålet skal vises for respondenten:

Dersom spørsmålet “Har du - de siste 12 månedene - hatt pustebesvær (hatt tungt for å puste, kjent deg tett i brystet, hatt pipende eller hvesende pust)?” inneholder noen av disse alternativene

- “Ja”

166) Har du - de siste 12 månedene - hatt pipende eller hvesende pust, tungt for å puste, eller besværlig hoste, i forbindelse med noe av det nedenstående?

- ☐ Nei, har ikke hatt besvær ved noe av dette
- ☐ Kald luft eller tåke
- ☐ Katt
- ☐ Hund
- ☐ Hest
- ☐ Bjørkepollen
- ☐ Gresspollen
- ☐ Burotpollen
- ☐ Psykisk belastning eller stress
- ☐ Tobakksrøyk
- ☐ Luftforurensninger
- ☐ Sterke dufter
- ☐ Mat eller matos

- [ ] Kald drikke
- [ ] Annet

Denne informasjonen vises kun i forhåndsvisningen

Følgende betingelser må være oppfylt for at spørsmålet skal vises for respondenten:

Dersom spørsmålet “Har du - de siste 12 månedene - hatt pustebesvær (hatt tungt for å puste, kjent deg tett i brystet, hatt pipende eller hvesende pust)?” inneholder noen av disse alternativene

- “Ja”

167) Har du - de siste 12 månedene - hatt pipende eller hvesende pust, tungt for å puste, eller besværlig hoste i forbindelse med anstrengelse?

- Nei
- Ja

168) Har du - de siste 12 månedene - brukt noen medisiner for astma eller pustebesvær?

○ Nei ○ Ja

Denne informasjonen vises kun i forhåndsvisningen

Følgende betingelser må være oppfylt for at spørsmålet skal vises for respondenten:

Dersom spørsmålet “Har du - de siste 12 månedene - brukt noen medisiner for astma eller pustebesvær?” inneholder noen av disse alternativene

- “Ja”

169) Hvilke medisiner for astma eller pustebesvær har du brukt de siste 12 månedene?

	Ved behov, eller for en kortere periode, noen uker av gangen	Over en lengre periode, minst 2 mnd
Bricanyl, Ventoline, Airomir, Buventol, Salbutamol Arrow	○	○
Pulmicort, Flutide, Becotide, Giona Easyhaler, Beklomet, AeroBec autohaler, Budesonid Arrow, Alvesco	○	○
Symbicort, Seretide	○	○
Oxis, Serevent, Onbrez Breezehaler	○	○
Atrovent, Ipraxa, Ipratropiumbromid	○	○
Singulair tabelett	○	○

Denne informasjonen vises kun i forhåndsvisningen

Følgende betingelser må være oppfylt for at spørsmålet skal vises for respondenten:

Dersom spørsmålet “Bricanyl, Ventoline, Airomir, Buventol, Salbutamol Arrow” inneholder noen av disse alternativene

- “Over en lengre periode, minst 2 mnd”
- “Ved behov, eller for en kortere periode, noen uker av gangen”

170) Dersom du bruker luftrørsutvidende medisin (Bricanyl, Ventoline, Airomir, buventol...), hvor ofte bruker du dem i løpet av en vanlig uke?

- Mindre enn 2 ganger pr uke
- 2 ganger eller mer pr uke

Denne informasjonen vises kun i forhåndsvisningen

Følgende betingelser må være oppfylt for at spørsmålet skal vises for respondenten:

Dersom spørsmålet “Har du - de siste 12 månedene - brukt noen medisiner for astma eller pustebesvær?” inneholder noen av disse alternativene

- “Ja”

171) Har du - de siste 12 månedene - tatt kortisontabletter oppløst i vann (f.eks Betapred) mot astma eller pustebesvær?

- Nei
- Ja

Denne informasjonen vises kun i forhåndsvisningen

Følgende betingelser må være oppfylt for at spørsmålet skal vises for respondenten:

Dersom spørsmålet “Har du - de siste 12 månedene - tatt kortisontabletter oppløst i vann (f.eks Betapred) mot astma eller pustebesvær?” inneholder noen av disse alternativene

- “Ja”

172) Har du tatt kortisontabletter oppløst i vann 3 dager i strekk eller mer de siste 12 månedene?

○ Nei ○ Ja

Denne informasjonen vises kun i forhåndsvisningen

Følgende betingelser må være oppfylt for at spørsmålet skal vises for respondenten:

Dersom spørsmålet “Har du - de siste 12 månedene - brukt noen medisiner for astma eller pustebesvær?” inneholder noen av disse alternativene

- “Ja”

173) Har du brukt medisiner for astma eller pustebesvær som er skrevet ut til andre?

- Nei
- Ja, delvis
- Ja, helt

Denne informasjonen vises kun i forhåndsvisningen

Følgende betingelser må være oppfylt for at spørsmålet skal vises for respondenten:

Dersom spørsmålet “Har du - de siste 12 månedene - brukt noen medisiner for astma eller pustebesvær?” inneholder noen av disse alternativene

- “Ja”

174) Har noen andre brukt dine medisiner for astma eller pustebesvær?

- Nei

- Ja, delvis
- Ja, helt

Denne informasjonen vises kun i forhåndsvisningen

Følgende betingelser må være oppfylt for at spørsmålet skal vises for respondenten:

Dersom spørsmålet “Har du - de siste 12 månedene - brukt noen medisiner for astma eller pustebesvær?” inneholder noen av disse alternativene

- “Ja”

175) Hvor mange inhalatorer av samme merke bruker du å ha samtidig? (men kanskje på ulike steder)

- 1 inhalator
- 2 inhalatorer
- 3 inhalatorer
- Mer enn 3 inhalatorer

Denne informasjonen vises kun i forhåndsvisningen

Følgende betingelser må være oppfylt for at spørsmålet skal vises for respondenten:

Dersom spørsmålet “Har du - de siste 12 månedene - brukt noen medisiner for astma eller pustebesvær?” inneholder noen av disse alternativene

- “Ja”

176) Hvor ofte hender det at du bruker din inhalator til den er tom?

- Aldri
- Sjelden
- Ofte
- Alltid

Denne informasjonen vises kun i forhåndsvisningen

Følgende betingelser må være oppfylt for at spørsmålet skal vises for respondenten:

Dersom spørsmålet “Har du - de siste 12 månedene - brukt noen medisiner for astma eller pustebesvær?” inneholder noen av disse alternativene

- “Ja”

177) Har du fått undervisning om din astmasykdom av sykepleier eller lege? (f.eks hva astma er slags sykdom, hvordan medisinene fungerer og hva du skal gjøre ved forverring)

- Nei
- Ja

178) Har du - de siste 12 månedene - hatt tørrhoste om natten uten samtidig å være forkjølet?

- Nei
- Ja

179) Har du vært plaget av hoste mesteparten av tiden, i minst 3 måneder pr år?

- Nei
- Ja

Denne informasjonen vises kun i forhåndsvisningen

Følgende betingelser må være oppfylt for at spørsmålet skal vises for respondenten:

Dersom spørsmålet “Har du vært plaget av hoste mesteparten av tiden, i minst 3 måneder pr år?” inneholder noen av disse alternativene

- “Ja”

180) Hvor mange år har du vært plaget med hoste mesteparten av tiden, i minst 3 måneder pr år?

- 1
- 2
- 3
- 4
- 5
- 6
- 7
- 8
- 9
- 10
- 11
- 12
- 13
- 14

- 15
- 16
- 17
- 18
- 19
- 20

181) Har du vært plaget av slim fra brystet mesteparten av tiden, i minst 3 måneder pr år?

- Nei
- Ja

Denne informasjonen vises kun i forhåndsvisningen

Følgende betingelser må være oppfylt for at spørsmålet skal vises for respondenten:

Dersom spørsmålet “Har du vært plaget av slim fra brystet mesteparten av tiden, i minst 3 måneder pr år?” inneholder noen av disse alternativene

- “Ja”

182) Hvor mange år har du vært plaget med slim fra brystet mesteparten av tiden, i minst 3 måneder pr år?

- 1
- 2
- 3
- 4
- 5
- 6
- 7
- 8
- 9
- 10
- 11
- 12
- 13
- 14

- 15
- 16
- 17
- 18
- 19
- 20

183) Om du har pustebesvær eller astma, har det...

	Ikke i det hele tatt	Litt	En del	Ganske mye	Mye
hindret deg i skolearbeidet	○	○	○	○	○
hindret deg i fritidsaktiviteter	○	○	○	○	○
uroet deg de siste 4 ukene	○	○	○	○	○

HUDPLAGER OG EKSEM

184) Har du noen gang vært plaget av kviser?

- Ja
- Nei
- Vet ikke

Denne informasjonen vises kun i forhåndsvisningen

Følgende betingelser må være oppfylt for at spørsmålet skal vises for respondenten:

Dersom spørsmålet “Har du noen gang vært plaget av kviser?” inneholder noen av disse alternativene
- “Ja”

185) Hvor mye plaget er du av kviser idag?

- 0 Ingen plager
- 1
- 2
- 3
- 4
- 5
- 6
- 7
- 8
- 9
- 10 Verst tenkelige plager

Denne informasjonen vises kun i forhåndsvisningen

Følgende betingelser må være oppfylt for at spørsmålet skal vises for respondenten:

Dersom spørsmålet “Har du noen gang vært plaget av kviser?” inneholder noen av disse alternativene

- “Ja”

186) Har du noen gang oppsøkt lege på grunn av kviser?

◯ Ja ◯ Nei

Denne informasjonen vises kun i forhåndsvisningen

Følgende betingelser må være oppfylt for at spørsmålet skal vises for respondenten:

Dersom spørsmålet “Har du noen gang oppsøkt lege på grunn av kviser?” inneholder noen av disse alternativene

- “Ja”

187) Har du fått noen av disse behandlingene av lege?

	Ja	Nei	Vet ikke
Lokalbehandling (f.eks. kremer eller oppløsninger)			
Antibiotika tabletter (f.eks. Tetracyclin)			
Roaccutan tabletter			

188) Har du eller har du noen gang hatt psoriasis?

Ja   Nei   Vet ikke

Denne informasjonen vises kun i forhåndsvisningen

Følgende betingelser må være oppfylt for at spørsmålet skal vises for respondenten:

Dersom spørsmålet “Har du eller har du noen gang hatt psoriasis?” inneholder noen av disse alternativene

- “Ja”

189) Hvor gammel var du første gang du fikk psoriasis?

- 10 år eller yngre
- 11 år
- 12 år
- 13 år
- 14 år
- 15 år
- 16 år
- 17 år
- 18 år
- 19 år
- 20 år
- 21 år
- 22 år
- 23 år
- 24 år

- 25 år
- 26 år
- 27 år
- 28 år
- 29 år
- 30 år eller eldre

Denne informasjonen vises kun i forhåndsvisningen

Følgende betingelser må være oppfylt for at spørsmålet skal vises for respondenten:

Dersom spørsmålet "Har du eller har du noen gang hatt psoriasis?" inneholder noen av disse alternativene
- "Ja"

190) Hvor mye plaget er du av psoriasis idag?

- 0 Ingen plager
- 1
- 2
- 3
- 4
- 5
- 6
- 7
- 8
- 9

- 10 Verst tenkelige plager

Verkebyller er svært store kviser som er ømme/smertefulle og som ofte gir arr.

191) Har du noen gang hatt verkebyller under armene/armhulene?

- Ja
- Nei
- Vet ikke

Denne informasjonen vises kun i forhåndsvisningen

Følgende betingelser må være oppfylt for at spørsmålet skal vises for respondenten:

Dersom spørsmålet “Har du noen gang hatt verkebyller under armene/armhulene?” inneholder noen av disse alternativene

- “Ja”

192) Har du noen gang oppsøkt lege på grunn av verkebyllene?

○ Ja ○ Nei

Denne informasjonen vises kun i forhåndsvisningen

Følgende betingelser må være oppfylt for at spørsmålet skal vises for respondenten:

Dersom spørsmålet “Har du noen gang hatt verkebyller under armene/armhulene?” inneholder noen av disse alternativene

- “Ja”

193) Har du noen gang hatt verkebyller i lyskene/nært skrittet?

- Ja
- Nei
- Vet ikke

Denne informasjonen vises kun i forhåndsvisningen

Følgende betingelser må være oppfylt for at spørsmålet skal vises for respondenten:

Dersom spørsmålet “Har du noen gang hatt verkebyller i lyskene/nært skrittet?” inneholder noen av disse alternativene

- “Ja”

194) Har du noen gang oppsøkt lege på grunn av verkebyllene?

- Ja
- Nei

195) Har du - de siste 12 månedene - hatt plager med tørr hud?

- Nei
- Ja

Denne informasjonen vises kun i forhåndsvisningen

Følgende betingelser må være oppfylt for at spørsmålet skal vises for respondenten:

Dersom spørsmålet “Har du - de siste 12 månedene - hatt plager med tørr hud?” inneholder noen av disse alternativene

- “Ja”

196) Har du - de siste 12 månedene - smurt deg med mykgjørende krem/lotion på grunn av tørr hud?

- Nei
- Ja, mindre enn 1 måned
- Ja, 1-6 måneder
- Ja, mer enn 6 måneder

197) Har du - de siste 12 månedene - hatt kløende utslett?

○ Nei ○ Ja

Denne informasjonen vises kun i forhåndsvisningen

Følgende betingelser må være oppfylt for at spørsmålet skal vises for respondenten:

Dersom spørsmålet “Har du - de siste 12 månedene - hatt kløende utslett?” inneholder noen av disse alternativene
- “Ja”

198) Hvor lenge pleier det kløende utslettet å vare?

- Mindre enn 1 uke
- 1-2 uker
- Mer enn 2 uker

Denne informasjonen vises kun i forhåndsvisningen

Følgende betingelser må være oppfylt for at spørsmålet skal vises for respondenten:

Dersom spørsmålet “Har du - de siste 12 månedene - hatt kløende utslett?” inneholder noen av disse alternativene
- “Ja”

199) Hvor har du de kløende utslettene? (Flere alternativer kan krysses av)

- I hodebunnen
- I ansiktet

- I ørene
- På halsen eller i nakken
- På håndledd eller fotledd
- På hendene
- På eller under rumpeballene
- På lårenes innsider
- På brystkasse, mage, rygg eller skuldre
- I armhulene
- På armenes eller benas utsider
- I albuebøyer eller knehaser
- I lysken eller underlivet
- På føttene
- Andre steder

Denne informasjonen vises kun i forhåndsvisningen

Følgende betingelser må være oppfylt for at spørsmålet skal vises for respondenten:

Dersom spørsmålet “Har du - de siste 12 månedene - hatt kløende utslett?” inneholder noen av disse alternativene
- “Ja”

200) Hvor gammel var du første gang du fikk denne typen utslett?

- 0 år
- 1 år
- 2 år

- 3 år
- 4 år
- 5 år
- 6 år
- 7 år
- 8 år
- 9 år
- 10 år
- 11 år
- 12 år
- 13 år
- 14 år
- 15 år
- 16 år
- 17 år
- 18 år eller mer

Denne informasjonen vises kun i forhåndsvisningen

Følgende betingelser må være oppfylt for at spørsmålet skal vises for respondenten:

Dersom spørsmålet “Har du - de siste 12 månedene - hatt kløende utslett?” inneholder noen av disse alternativene
- “Ja”

201) I hvilken periode i løpet av året har du hatt kløende utslett de siste 12 månedene?

- Januar
- Februar
- Mars
- April
- Mai
- Juni
- Juli
- August
- September
- Oktober
- November
- Desember

Denne informasjonen vises kun i forhåndsvisningen

Følgende betingelser må være oppfylt for at spørsmålet skal vises for respondenten:

Dersom spørsmålet “Har du - de siste 12 månedene - hatt kløende utslett?” inneholder noen av disse alternativene
- “Ja”

202) Har det kløende utslettet forsvunnet helt ved noe tidspunkt de siste 12 månedene?

○ Nei ○ Ja

Denne informasjonen vises kun i forhåndsvisningen

Følgende betingelser må være oppfylt for at spørsmålet skal vises for respondenten:

Dersom spørsmålet “Har du - de siste 12 månedene - hatt kløende utslett?” inneholder noen av disse alternativene
- “Ja”

203) Har du - de siste 12 månedene - hatt vanskelig for å få sove, eller våknet pga kløende utslett?

○ Nei

○ Mindre enn 1 gang pr uke

○ 1 eller flere ganger pr uke

Denne informasjonen vises kun i forhåndsvisningen

Følgende betingelser må være oppfylt for at spørsmålet skal vises for respondenten:

Dersom spørsmålet “Har du - de siste 12 månedene - hatt kløende utslett?” inneholder noen av disse alternativene
- “Ja”

204) Har du - den siste uken - hatt kløende utslett?

○ Nei ○ Ja

Denne informasjonen vises kun i forhåndsvisningen

Følgende betingelser må være oppfylt for at spørsmålet skal vises for respondenten:

Dersom spørsmålet “Har du - den siste uken - hatt kløende utslett?” inneholder noen av disse alternativene
- “Ja”

205) I løpet av den siste uken, hvor mye har huden din klødd eller føltes smertefull?

- Veldig mye
- Ganske mye
- Litt
- Ikke i det hele tatt

Denne informasjonen vises kun i forhåndsvisningen

Følgende betingelser må være oppfylt for at spørsmålet skal vises for respondenten:

Dersom spørsmålet “Har du - den siste uken - hatt kløende utslett?” inneholder noen av disse alternativene
- “Ja”

206) I løpet av den siste uken, hvor plaget, trist eller lei deg, har du vært pga huden?

- Veldig mye
- Ganske mye
- Litt
- Ikke i det hele tatt

Denne informasjonen vises kun i forhåndsvisningen

Følgende betingelser må være oppfylt for at spørsmålet skal vises for respondenten:

Dersom spørsmålet “Har du - den siste uken - hatt kløende utslett?” inneholder noen av disse alternativene

- “Ja”

207) I løpet av den siste uken, har huden din påvirket hvordan det har vært å være sammen med dine venner?

- Veldig mye
- Ganske mye
- Litt
- Ikke i det hele tatt

Denne informasjonen vises kun i forhåndsvisningen

Følgende betingelser må være oppfylt for at spørsmålet skal vises for respondenten:

Dersom spørsmålet “Har du - den siste uken - hatt kløende utslett?” inneholder noen av disse alternativene

- “Ja”

208) I løpet av den siste uken, har du byttet eller hatt på deg andre eller spesielle klær/sko på grunn av din hud?

- Veldig mye
- Ganske mye
- Litt
- Ikke i det hele tatt

Denne informasjonen vises kun i forhåndsvisningen

Følgende betingelser må være oppfylt for at spørsmålet skal vises for respondenten:

Dersom spørsmålet “Har du - den siste uken - hatt kløende utslett?” inneholder noen av disse alternativene

- “Ja”

209) I løpet av den siste uken, har dine hudplager påvirket deg når det gjelder å gå ut eller holde på med dine hobbyer?

- Veldig mye
- Ganske mye
- Litt
- Ikke i det hele tatt

Denne informasjonen vises kun i forhåndsvisningen

Følgende betingelser må være oppfylt for at spørsmålet skal vises for respondenten:

Dersom spørsmålet “Har du - den siste uken - hatt kløende utslett?” inneholder noen av disse alternativene

- “Ja”

210) I løpet av den siste uken, har du unngått svømming eller annen trening pga dine hudplager?

- Veldig mye
- Ganske mye
- Litt
- Ikke i det hele tatt

Denne informasjonen vises kun i forhåndsvisningen

Følgende betingelser må være oppfylt for at spørsmålet skal vises for respondenten:

Dersom spørsmålet “Har du - den siste uken - hatt kløende utslett?” inneholder noen av disse alternativene

- “Ja”

211) I løpet av den siste uken, har huden din påvirket ditt skolearbeid?

- Veldig mye
- Ganske mye
- Litt
- Ikke i det hele tatt

Denne informasjonen vises kun i forhåndsvisningen

Følgende betingelser må være oppfylt for at spørsmålet skal vises for respondenten:

Dersom spørsmålet “Har du - den siste uken - hatt kløende utslett?” inneholder noen av disse alternativene

- “Ja”

212) Dersom du har hatt ferie: I løpet av den siste uken, har dine hudplager hindret deg i å nyte ferien?

- Veldig mye

- Ganske mye
- Litt
- Ikke i det hele tatt

Denne informasjonen vises kun i forhåndsvisningen

Følgende betingelser må være oppfylt for at spørsmålet skal vises for respondenten:

Dersom spørsmålet “Har du - den siste uken - hatt kløende utslett?” inneholder noen av disse alternativene

- “Ja”

213) I løpet av den siste uken, hvor mye plager har du hatt pga din hud fordi andre personer har gitt deg tilnavn, ertet deg, mobbet deg, stilt spørsmål eller unngått deg?

- Veldig mye
- Ganske mye
- Litt
- Ikke i det hele tatt

Denne informasjonen vises kun i forhåndsvisningen

Følgende betingelser må være oppfylt for at spørsmålet skal vises for respondenten:

Dersom spørsmålet “Har du - den siste uken - hatt kløende utslett?” inneholder noen av disse alternativene

- “Ja”

214) I løpet av den siste uken, hvor mye har din søvn blitt påvirket av dine hudplager?

- Veldig mye
- Ganske mye
- Litt
- Ikke i det hele tatt

Denne informasjonen vises kun i forhåndsvisningen

Følgende betingelser må være oppfylt for at spørsmålet skal vises for respondenten:

Dersom spørsmålet “Har du - den siste uken - hatt kløende utslett?” inneholder noen av disse alternativene

- “Ja”

215) I løpet av den siste uken, hvor mye problem har du hatt med behandlingen av huden din?

- Veldig mye
- Ganske mye
- Litt
- Ikke i det hele tatt

216) Har du - de siste 12 månedene - hatt eksem?

○ Nei ○ Ja

Denne informasjonen vises kun i forhåndsvisningen

Følgende betingelser må være oppfylt for at spørsmålet skal vises for respondenten:

Dersom spørsmålet “Har du - de siste 12 månedene - hatt eksem?” inneholder noen av disse alternativene

- “Ja”

217) Hvor lenge har du tilsammen hatt eksem de siste 12 månedene?

- Mindre enn 1 måned
- 1-3 måneder
- 4-6 måneder
- Mer enn 6 måneder

Denne informasjonen vises kun i forhåndsvisningen

Følgende betingelser må være oppfylt for at spørsmålet skal vises for respondenten:

Dersom spørsmålet “Har du - de siste 12 månedene - hatt eksem?” inneholder noen av disse alternativene

- “Ja”

218) Har du smurt deg med kortison pga eksem de siste 12 månedene?

- Nei
- Ja, mindre enn 1 måned

- ○ Ja, 1-6 måneder
- ○ Ja, mer enn 6 måneder

219) Har du noen gang hatt håndeksem? (Kløende forandring i huden, blemmer eller kløende utslett)

- Nei
- Ja

Denne informasjonen vises kun i forhåndsvisningen

Følgende betingelser må være oppfylt for at spørsmålet skal vises for respondenten:

Dersom spørsmålet “Har du noen gang hatt håndeksem? (Kløende forandring i huden, blemmer eller kløende utslett)” inneholder noen av disse alternativene

- “Ja”

220) Hvor gammel var du da håndeksemet begynte?

- 0 år
- 1 år
- 2 år
- 3 år
- 4 år
- 5 år
- 6 år
- 7 år
- 8 år
- 9 år
- 10 år
- 11 år
- 12 år
- 13 år
- 14 år

- 15 år
- 16 år
- 17 år
- 18 år
- 19 år
- 20 år

Denne informasjonen vises kun i forhåndsvisningen

Følgende betingelser må være oppfylt for at spørsmålet skal vises for respondenten:

Dersom spørsmålet “Har du noen gang hatt håndeksem? (Kløende forandring i huden, blemmer eller kløende utslett)” inneholder noen av disse alternativene
- “Ja”

221) Har du - de siste 12 månedene - ved noen anledning hatt håndeksem?

- Nei
- Ja

Denne informasjonen vises kun i forhåndsvisningen

Følgende betingelser må være oppfylt for at spørsmålet skal vises for respondenten:

Dersom spørsmålet “Har du - de siste 12 månedene - ved noen anledning hatt håndeksem?” inneholder noen av disse alternativene

- “Ja”

222) Hvor mye plaget er du av håndeksem i dag?

- 0 Ingen plager
- 1
- 2
- 3
- 4
- 5
- 6
- 7
- 8
- 9
- 10 Verst tenkelige plager

223) Hvor mange ganger kommer hendene dine i kontakt med vann i løpet av en dag? (ikke tell med den tiden du beskytter hendene med hansker)

- Ingen ganger pr dag
- 1-10 ganger pr dag
- 11-20 ganger pr dag
- 21-30 ganger pr dag
- Mer enn 30 ganger pr dag

224) Har du - noen gang - fått kløende utslett eller eksem (rødhet, blemmer eller flassing) av sminke eller hygieneprodukter?

- Nei
- Ja

Denne informasjonen vises kun i forhåndsvisningen

Følgende betingelser må være oppfylt for at spørsmålet skal vises for respondenten:

Dersom spørsmålet “Har du - noen gang - fått kløende utslett eller eksem (rødhet, blemmer eller flassing) av sminke eller hygieneprodukter?” inneholder noen av disse alternativene

- “Ja”

225) Av hva har du fått plager?

- ☐ Sminke eller parfyme
- ☐ Deodorant
- ☐ Shampo eller balsam
- ☐ Såpe eller dusjkrem
- ☐ Annet

226) Har du - noen gang - farget håret? (farget, tonet, bleket eller stripet håret ditt, hjemme eller hos frisør)

○ Nei ○ Ja

Denne informasjonen vises kun i forhåndsvisningen

Følgende betingelser må være oppfylt for at spørsmålet skal vises for respondenten:

Dersom spørsmålet “Har du - noen gang - farget håret? (farget, tonet, bleket eller stripet håret ditt, hjemme eller hos frisør)” inneholder noen av disse alternativene

- “Ja”

227) Har du noen gang fått plager ved hårfarging?

○ Nei ○ Ja

Denne informasjonen vises kun i forhåndsvisningen

Følgende betingelser må være oppfylt for at spørsmålet skal vises for respondenten:

Dersom spørsmålet “Har du noen gang fått plager ved hårfarging?” inneholder noen av disse alternativene

- “Ja”

228) På hvilken måte har du reagert når du har farget håret?

- ☐ Reaksjon i ansiktet, hodebunn, på ørene eller halsen (rødhet, flassing, kløe)
- ☐ Kraftig reaskjon i ansiktet, hodebunn, på ørene eller halsen (hevelse, væskende utslett)
- ☐ Reaksjon på hendene (rødhet, flassing, kløe)
- ☐ Annet

229) Har du - noen gang - fått kløende utslett eller eksem (rødhet, blemmer eller flassing) av latex eller gummi (ballonger, gummihansker, kondomer...)?

- Nei
- Ja

Denne informasjonen vises kun i forhåndsvisningen

Følgende betingelser må være oppfylt for at spørsmålet skal vises for respondenten:

Dersom spørsmålet “Har du - noen gang - fått kløende utslett eller eksem (rødhet, blemmer eller flassing) av latex eller gummi (ballonger, gummihansker, kondomer...)?” inneholder noen av disse alternativene

- “Ja”

230) Av hvilke latex-/gummiprodukter har du fått plager?

- ☐ Ballonger ved ballongblåsing
- ☐ Gummihansker
- ☐ Kondomer
- ☐ Annet

231) Har du - noen gang - tatt hull i ørene eller laget hull for smykker noen andre steder på kroppen?

○ Nei ○ Ja

232) Har du noen tatovering?

○ Nei ○ Ja

Denne informasjonen vises kun i forhåndsvisningen

Følgende betingelser må være oppfylt for at spørsmålet skal vises for respondenten:

Dersom spørsmålet “Har du noen tatovering?” inneholder noen av disse alternativene

- “Ja”

233) Har du noen gang fått kløende utslett eller eksem (rødhet, blemmer eller flassing) av din tatovering?

◯ Nei ◯ Ja

234) Har du - noen gang - fått kløende utslett eller eksem (rødhet, blemmer eller flassing) av metallgjenstander?

○ Nei ○ Ja

Denne informasjonen vises kun i forhåndsvisningen

Følgende betingelser må være oppfylt for at spørsmålet skal vises for respondenten:

Dersom spørsmålet “Har du - noen gang - fått kløende utslett eller eksem (rødhet, blemmer eller flassing) av metallgjenstander?” inneholder noen av disse alternativene

- “Ja”

235) Av hva har du fått kløende utslett eller eksem?

- ☐ Armbånd, halsbånd, fingerring eller annet
- ☐ Smykke (unntatt smykker for hull)
- ☐ Smykker for hull
- ☐ Knapp, nål, spenne, glidelås eller liknende i sko eller støvler
- ☐ Klokke eller klokkereim
- ☐ Briller eller solbriller
- ☐ Hårspenner eller liknende
- ☐ Mobiltelefon
- ☐ Øretelefon
- ☐ Annet

236) Dersom du har hudbesvær eller eksem, har det...

	Ikke i det hele tatt	Litt	En del	Ganske mye	Mye
Hindret deg i skolearbeidet	○	○	○	○	○
Hindret deg i fritidsaktiviteter	○	○	○	○	○
Bekymret (Uroet) deg de siste fire ukene	○	○	○	○	○

NESE- ELLER ØYEPLAGER

237) Har du - de siste 12 månedene - hatt nysing, kløende nese, rennende nese eller tett nese uten at du samtidig har vært forkjølet?

○ Nei ○ Ja

Denne informasjonen vises kun i forhåndsvisningen

Følgende betingelser må være oppfylt for at spørsmålet skal vises for respondenten:

Dersom spørsmålet “Har du - de siste 12 månedene - hatt nysing, kløende nese, rennende nese eller tett nese uten at du samtidig har vært forkjølet?” inneholder noen av disse alternativene

- “Ja”

238) Har du hatt nysing, kløende nese, rennende nese eller tett nese i mer en 4 dager uten at du samtidig har vært forkjølet i de siste 12 månedene?

○ Nei ○ Ja

Denne informasjonen vises kun i forhåndsvisningen

Følgende betingelser må være oppfylt for at spørsmålet skal vises for respondenten:

Dersom spørsmålet “Har du hatt nysing, kløende nese, rennende nese eller tett nese i mer en 4 dager uten at du samtidig har vært forkjølet i de siste 12 månedene?” inneholder noen av disse alternativene

- “Ja”

239) Skjedde dette over 4 uker i strekk de siste 12 månedene?

○ Nei ○ Ja

Denne informasjonen vises kun i forhåndsvisningen

Følgende betingelser må være oppfylt for at spørsmålet skal vises for respondenten:

Dersom spørsmålet “Har du hatt nysing, kløende nese, rennende nese eller tett nese i mer en 4 dager uten at du samtidig har vært forkjølet i de siste 12 månedene?” inneholder noen av disse alternativene

- “Ja”

240) Hvor lenge har du hatt disse plagene uten samtidig å være forkjølet de siste 12 månedene?

○ Mindre enn 1 måned

- 1-3 måneder
- 3-6 måneder
- Mer enn 6 måneder

Denne informasjonen vises kun i forhåndsvisningen

Følgende betingelser må være oppfylt for at spørsmålet skal vises for respondenten:

Dersom spørsmålet “Har du - de siste 12 månedene - hatt nysing, kløende nese, rennende nese eller tett nese uten at du samtidig har vært forkjølet?” inneholder noen av disse alternativene

- “Ja”

241) Har disse neseplagene - de siste 12 månedene - forekommet samtidig med kløende, rennende øyne?

○ Nei ○ Ja

Denne informasjonen vises kun i forhåndsvisningen

Følgende betingelser må være oppfylt for at spørsmålet skal vises for respondenten:

Dersom spørsmålet “Har du - de siste 12 månedene - hatt nysing, kløende nese, rennende nese eller tett nese uten at du samtidig har vært forkjølet?” inneholder noen av disse alternativene

- “Ja”

242) I løpet av hvilken periode har du hatt plager med nysing, kløende nese, rennende nese eller tett nese de siste 12 månedene?

☐ Januar

☐ Februar

- Mars
- April
- Mai
- Juni
- Juli
- August
- September
- Oktober
- November
- Desember

243) Har du hatt nese- eller øyeplager, uten å være forkjølet, ved kontakt med noe av det nedenstående de siste 12 månedene?

- ☐ Nei, har ikke hatt besvær med noen av disse
- ☐ Katt
- ☐ Hund
- ☐ Hest
- ☐ Kanin, marsvin eller andre gnagere
- ☐ Bjørkepollen
- ☐ Gresspollen
- ☐ Burotpollen
- ☐ Tobakksrøyk
- ☐ Luftforurensninger
- ☐ Sterke dufter
- ☐ Annet

244) Har du unnlatt å gjøre ting du har villet gjøre pga neseplager de siste 12 månedene?

○ Nei ○ Ja

Denne informasjonen vises kun i forhåndsvisningen

Følgende betingelser må være oppfylt for at spørsmålet skal vises for respondenten:

Dersom spørsmålet “Har du unnlatt å gjøre ting du har villet gjøre pga neseplager de siste 12 månedene?” inneholder noen av disse alternativene

- “Ja”

245) Hvor mye har neseplagene påvirket at du har unnlatt å gjøre ting du har villet gjøre de siste 12 månedene?

- Litt
- Moderat
- Ganske mye
- Mye

246) Har du hatt vanskelig for å sove pga neseplager de siste 12 månedene?

Nei Ja

247) Har du - de siste 12 månedene - tatt noen medisiner for allergisnue/høysnue?

Nei Ja

Denne informasjonen vises kun i forhåndsvisningen

Følgende betingelser må være oppfylt for at spørsmålet skal vises for respondenten:

Dersom spørsmålet “Har du - de siste 12 månedene - tatt noen medisiner for allergisnue/høysnue?” inneholder noen av disse alternativene

- “Ja”

248) Hvilke medisiner for allergisnue/høysnue har du brukt de siste 12 månedene?

- ☐ Øyedråper
- ☐ Nesespray
- ☐ Allergitabletter
- ☐ Andre

249) Dersom du har hatt neseplager, allergisnue/høysnue, hvor plagsomt opplevde du at det var de siste 12 månedene?

- 0 Ingen plager
- 1
- 2
- 3
- 4
- 5
- 6
- 7
- 8
- 9
- 10 Verst tenkelige plager

250) Har du - de siste 12 månedene - vært tett i nesen?

- Nei
- Mindre enn 10 dager
- 10 dager - 12 uker
- 12 uker eller mer

251) Har du - de siste 12 månedene - hatt gulgrønt slim eller snørr bak i halsen?

- Nei
- Mindre enn 10 dager
- 10 dager - 12 uker
- 12 uker eller mer

252) Har du - de siste 12 månedene - hatt nedsatt luktesans?

- Nei
- Mindre enn 10 dager
- 10 dager - 12 uker
- 12 uker eller mer

253) Har du - de siste 12 månedene - opplevd smerter eller trykk ved eller omkring pannen, nesen eller øynene?

- Nei
- Mindre enn 10 dager
- 10 dager - 12 uker
- 12 uker eller mer

Denne informasjonen vises kun i forhåndsvisningen

Følgende betingelser må være oppfylt for at spørsmålet skal vises for respondenten:

Dersom spørsmålet “Har du - de siste 12 månedene - vært tett i nesen?” inneholder noen av disse alternativene

- “12 uker eller mer”
- “10 dager - 12 uker”
- “Mindre enn 10 dager”

eller

Dersom spørsmålet “Har du - de siste 12 månedene - hatt gulgrønt slim eller snørr bak i halsen?” inneholder noen av disse alternativene

- “12 uker eller mer”
- “10 dager - 12 uker”
- “Mindre enn 10 dager”

eller

Dersom spørsmålet “Har du - de siste 12 månedene - hatt nedsatt luktesans?” inneholder noen av disse alternativene

- “12 uker eller mer”
- “10 dager - 12 uker”
- “Mindre enn 10 dager”

eller

Dersom spørsmålet “Har du - de siste 12 månedene - opplevd smerter eller trykk ved eller omkring pannen, nesen eller øynene?” inneholder noen av disse alternativene

- “12 uker eller mer”
- “10 dager - 12 uker”
- “Mindre enn 10 dager”

254) Dersom du har hatt nesetetthet, snue, nedsatt luktesans eller smerter i ansiktet, hvor plagsomt synes du det var de siste 12 månedene?

- 0 Ikke plagsomt i det hele tatt
- 1
- 2
- 3
- 4
- 5
- 6
- 7
- 8
- 9
- 10 Verst tenkelig

255) Dersom du har hatt nese- eller øyeplager, har det...

	Ikke i det hele tatt	Litt	En del	Ganske mye	Mye
Hindret deg i skolearbeid	○	○	○	○	○
Hindret deg i fritidsaktiviteter	○	○	○	○	○
Bekymret deg de siste 4 uker	○	○	○	○	○

Reaksjoner på mat

256) Har du - de siste 12 månedene - reagert på noe i maten?

○ Nei ○ Ja

Denne informasjonen vises kun i forhåndsvisningen

Følgende betingelser må være oppfylt for at spørsmålet skal vises for respondenten:

Dersom spørsmålet “Har du - de siste 12 månedene - reagert på noe i maten?” inneholder noen av disse alternativene

- “Ja”

257) Har du reagert på noen av de nedenstående matvarene de siste 12 månedene?

- Melk - protein
- Melk - laktose
- Egg
- Fisk
- Skalldyr
- Hvete, andre kornslag
- Soya
- Sesam
- Eple, pære
- Fersken, nektarin, plommer, kirsebær
- Kiwi
- Banan
- Rå gulrot
- Peanøtter
- Hasselnøtter

- ☐ Mandel
- ☐ Valnøtt, pekannøtt
- ☐ Cashewnøtt, pistasjnøtt
- ☐ Paranøtt
- ☐ Annet

Denne informasjonen vises kun i forhåndsvisningen

Følgende betingelser må være oppfylt for at spørsmålet skal vises for respondenten:

Dersom spørsmålet “Har du reagert på noen av de nedenstående matvarene de siste 12 månedene?” inneholder noen av disse alternativene

- “Fisk”

258) Dersom du reagerer på fisk, hvilke(n) reaksjon(er) får du?

- [ ] Kløende utslett
- [ ] Hevelse i og rundt munnen
- [ ] Hevelse i ansiktet
- [ ] Slim i halsen
- [ ] Oppkast
- [ ] Diare
- [ ] Tungpust
- [ ] Svimmelhet
- [ ] Besvimelse/allergisjokk

Denne informasjonen vises kun i forhåndsvisningen

Følgende betingelser må være oppfylt for at spørsmålet skal vises for respondenten:

Dersom spørsmålet “Har du reagert på noen av de nedenstående matvarene de siste 12 månedene?” inneholder noen av disse alternativene

- “Fisk”

259) Dersom du reagerer på fisk, reagerer du ved å...

- [ ] Spise fisk
- [ ] Ta på fisk
- [ ] Puste inn damp fra fisk som kokes eller stekes

260) Dersom du ikke reagerer på fisk nå, har du:

- Reagert på fisk tidligere
- Aldri reagert på fisk

Denne informasjonen vises kun i forhåndsvisningen

Følgende betingelser må være oppfylt for at spørsmålet skal vises for respondenten:

Dersom spørsmålet “Dersom du ikke reagerer på fisk nå, har du:” inneholder noen av disse alternativene

- “Reagert på fisk tidligere”

261) Dersom du tidligere har reagert på fisk, hvilke(n) reaksjon(er) fikk du da?

- [ ] Kløende utslett
- [ ] Hevelse i og rundt munnen
- [ ] Hevelse i ansiktet
- [ ] Slim i halsen
- [ ] Oppkast
- [ ] Diare
- [ ] Tungpust
- [ ] Svimmelhet
- [ ] Besvimelse/allergisjokk

Denne informasjonen vises kun i forhåndsvisningen

Følgende betingelser må være oppfylt for at spørsmålet skal vises for respondenten:

Dersom spørsmålet “Har du - de siste 12 månedene - reagert på noe i maten?” inneholder noen av disse alternativene

- “Ja”

262) Har du en adrenalinsprøyte (Epipen, Anapen, Jext) som du kan ta, dersom du reagerer på noe i maten?

○ Nei ○ Ja

Denne informasjonen vises kun i forhåndsvisningen

Følgende betingelser må være oppfylt for at spørsmålet skal vises for respondenten:

Dersom spørsmålet “Har du en adrenalinsprøyte (Epipen, Anapen, Jext) som du kan ta, dersom du reagerer på noe i maten?” inneholder noen av disse alternativene

- “Ja”

263) Har du - noen gang - brukt sprøyten?

○ Nei ○ Ja

Denne informasjonen vises kun i forhåndsvisningen

Følgende betingelser må være oppfylt for at spørsmålet skal vises for respondenten:

Dersom spørsmålet “Har du en adrenalinsprøyte (Epipen, Anapen, Jext) som du kan ta, dersom du reagerer på noe i maten?” inneholder noen av disse alternativene

- “Ja”

264) Har du - de siste 12 månedene - brukt sprøyten?

○ Nei ○ Ja

Denne informasjonen vises kun i forhåndsvisningen

Følgende betingelser må være oppfylt for at spørsmålet skal vises for respondenten:

Dersom spørsmålet “Har du - de siste 12 månedene - reagert på noe i maten?” inneholder noen av disse alternativene

- “Ja”

265) Dersom du får plager av matvarer, har det...

	Ikke i det hele tatt	Litt	En del	Ganske mye	Mye
Hindret deg i skolearbeid	○	○	○	○	○
Hindret deg i fritidsaktiviteter	○	○	○	○	○
Bekymret deg de siste 4 ukene	○	○	○	○	○

Send

100 % fullført

© Copyright www.questback.com. All Rights Reserved.
