# Supplementary material for: Hormonal contraceptive use and Staphylococcus aureus nasal and throat carriage in a Norwegian youth population
Source: PLoS One. 2019 Jul 5;14(7):e0218511. doi: 10.1371/journal.pone.0218511 (PMC6611591; doi:10.1371/journal.pone.0218511)
Supplement: S2 Text — Interview from TFF2 in original language. (DOC) [file pone.0218511.s004.doc]

**Fit futures II**

**Tromsøundersøkelsen**

**INTERVJU ved frammøte**

GUL MARKERING: Endringer i Fit futures jamfør Protokoll datert 03092010

**Sykdom og medisiner:**

Har du noen form for kronisk (somatisk) sykdom /diagnose? eventuelt hvilke

Har du eksem nå?

Har du hatt vondt i halsen eller vært forkjølet i løpet av den siste måneden?

Hvordan er din helsetilstand i dag (feber, luftveisinfeksjon/ forkjølelse)?

Bruker du noen faste medisiner? eventuelt hvilke

Har du brukt antibiotika siste 24 t? eventuelt hvilke

Har du brukt antibiotika siste 3 måneder? eventuelt hvilke

Har du brukt smertestillende siste 24 t? eventuelt hvilke

Har du fått vaksine mot smittsom hjernehinnebetennelse /meningokokkvaksine som er anbefalt for russ?

Hvis Ja, angi dato for vaksinasjon. (omtrentlig dato er ok)

**Tid siden siste måltid:**

**KVINNEHELSE:**

Alder ved menarche

Dato siste menstruasjon

Sykluslengde og regelmessighet

Hormonelle prevensjonsmidler: p-piller/p-sprøyte/p-stav/p-plaster/p-ring/hormonspiral

**Graviditet**

**Er det noen mulighet for at du kan være gravid nå?**

⁪ Ja ⁪ Nei

Hvis ja:

**Er det greit for deg at vi tar en gravitest?**

⁪ Ja ⁪ Nei

(resultat av prøven formidles ikke til foreldre)

Hvis ja:

**Resultat av gravitest:**

⁪ Negativ ⁪ Positiv ⁪ Ikke utført

**Klarert for DEXA (genereres automatisk)**

⁪ Ja ⁪ Nei

*Følgende personer er ikke klarert:*

*Kvinner som sier det er mulighet for at de er gravide som ikke vil gjøre gravitest*

*Kvinner som har positiv gravitest.*

**NETTVERK**

Hvilke elever på trinn 3 har du hatt mest kontakt med den siste uka? Nevn inntil 5 elever, ved din egen skole eller andre skoler i Tromsø og Balsfjord.

For hver av de 5:

Hadde dere fysisk kontakt, for eksempel med håndtrykk, klem eller lignende.

Ja

Nei

Hvor hadde dere kontakt?

1. skolen
2. idrett
3. hjemmebesøk
4. annet

I hvilken grad synes du tabellen gir en oversikt over ditt sosiale nettverk? (Marker på tallrekken)

I liten grad I middels grad I stor grad

0 1 2 3 4 5 6 7 8 9 10
